# Respiratory Tract Infections in Patients With Inflammatory Bowel Disease: Safety Analyses From Vedolizumab Clinical Trials

**DOI:** 10.1093/ecco-jcc/jjy047

**Published:** 2018-05-17

**Authors:** Brian G Feagan, Fatima Bhayat, Mona Khalid, Aimee Blake, Simon P L Travis

**Affiliations:** 1 Robarts Clinical Trials, Robarts Research Institute, University of Western Ontario, London, ON, Canada; 2 Global Patient Safety, Takeda Pharmaceuticals International Co., Cambridge, MA, USA; 3 Evidence and Value generation, Takeda International – UK Branch, London, UK; 4 Translational Gastroenterology Unit, John Radcliffe Hospital, Oxford, UK

**Keywords:** α4β7 integrin, adverse event, clinical trials, bronchopneumonia, Crohn’s disease, GEMINI 1, GEMINI 2, gut lymphocyte trafficking, hospitalization, humanised monoclonal antibody, induction therapy, inflammatory bowel disease, lower respiratory tract infection, long-term safety, maintenance therapy, nasopharyngitis, placebo, pneumonia, post hoc analysis, respiratory tract infection, smoking status, systemic immunosuppressive therapy, tumour necrosis factor antagonist, ulcerative colitis; upper respiratory tract infectionl, vedolizumab

## Abstract

**Background and Aims:**

Vedolizumab, a humanised monoclonal antibody for the treatment of inflammatory bowel disease, selectively blocks gut lymphocyte trafficking. This may reduce the risk of respiratory tract infections [RTIs] compared with systemic immunosuppressive therapies. To assess this possibility, we evaluated the rates of RTIs in clinical trials of vedolizumab.

**Methods:**

Patient-level data from Phase 3 randomised controlled trials [RCTs] of vedolizumab in ulcerative colitis [UC; GEMINI 1] and Crohn’s disease [CD; GEMINI 2], and a long-term safety study [UC and CD] were pooled. Cox proportional hazards models were used to estimate the incidence of upper RTIs [URTIs] and lower RTIs [LRTIs] with adjustment for significant covariates.

**Results:**

In the RCTs [*n =* 1731 patients], the incidence of URTIs was numerically higher in patients receiving vedolizumab compared with those receiving placebo, although this difference was not statistically significant (38.7 vs 33.0 patients per 100 patient-years; hazard ratio [HR] 1.12; 95% confidence interval [CI]: 0.83–1.51; *p =* 0.463). The rate of LRTIs, including pneumonia, was numerically lower in the vedolizumab versus the placebo group: this difference was not statistically significant (7.7 vs 8.5 per 100 patient-years [HR 0.85; 95% CI: 0.48–1.52; *p =* 0.585]). Both URTIs and LRTIs were more frequent in patients with CD compared with UC. Most RTIs in patients receiving vedolizumab were not serious and did not require treatment discontinuation.

**Conclusions:**

Vedolizumab therapy was not associated with an increased incidence of respiratory tract infection compared with placebo.

## 1. Introduction

Tumour necrosis factor [TNF] antagonists have revolutionised the treatment of inflammatory bowel disease [IBD]. Although these drugs are considered relatively safe, the increased risk of serious infection remains an important concern.^1–4^ Upper respiratory tract infections [URTIs] are among the most common adverse events [AEs] reported in randomised controlled trials of TNF antagonists,^5–8^ and an increased incidence of URTIs in comparison with placebo has been consistently observed in these studies.^7,9,10^

Patients with IBD have an increased risk of pneumonia which is further intensified by TNF antagonist therapy.^11^ In a retrospective cohort study, the risk of developing pneumonia was approximately 50% higher for patients with IBD [*n =* 108604] than for the general population (*n =* 434416; hazard ratio [HR] 1.54; 95% confidence interval [CI]: 1.49–1.60), and among those with IBD, TNF antagonist therapy was independently associated with pneumonia (odds ratio [OR] 1.28; 95% CI: 1.08–1.52).^11^ In a US hospitalisation database, 27.5% of all Crohn’s disease [CD]- or ulcerative colitis [UC]-related hospitalisations were attributable to infection.^12^ These patients had excess mortality risks compared with patients without infection-related hospitalisations, which varied depending on the infection type; pneumonia had one of the highest excess mortality risks compared with patients without infection-related hospitalisation [OR 3.6; 95% CI: 2.9–4.5]. An analysis of the TREAT™ registry, which evaluated 6273 patients with CD over a mean follow-up of 5.2 years, found that infliximab therapy was independently associated with an increased risk of serious infection (2.06 events per 100 patient-years [HR 1.43; 95% CI: 1.11–1.84; *p =* 0.006]).^3^ The most common serious infection was pneumonia, with an incidence of 0.24 cases per 100 patient-years of follow-up in infliximab-treated patients compared with a rate of 0.14 cases per 100 patient-years of follow-up for those on treatments other than infliximab.^3^ Similarly, in the ENCORE registry, which followed 1541 infliximab-treated patients with CD for up to 5 years, infliximab therapy was independently associated with an increased risk of serious infection [HR 1.64; 95% CI: 1.17–2.31], and the most common AEs were abscess and pneumonia.^13^

Vedolizumab, a humanised monoclonal antibody that binds to the α_4_β_7_ integrin and selectively blocks lymphocyte trafficking to the gut,^14,15^ has been shown to be an effective induction and maintenance therapy for both UC and CD.^16,17^ Unlike thiopurines, methotrexate, and TNF antagonists, the gut-selective mechanism of action of vedolizumab^15^ does not result in systemic immunosuppression.^14^ Although a previous evaluation of the safety of vedolizumab did not identify an increased risk of respiratory tract infection [RTI],^18^ the estimates used in that study were based on simple incidence rates and were not adjusted for important covariates such as smoking status and age. Given the previously described increase in risk associated with TNF antagonists, it is important to accurately estimate the incidence of RTIs in patients treated with vedolizumab. We used an integrated dataset derived from placebo-controlled trials to obtain such estimates.

## 2. Materials and Methods

### 2.1. Data sources: GEMINI 1 and 2 studies and the GEMINI open-label extension

Data from two Phase 3, randomised, placebo-controlled clinical trials of vedolizumab, GEMINI 1 [UC] and GEMINI 2 [CD], were analysed for rates of URTIs and LRTIs. GEMINI 1 and 2 evaluated the efficacy and safety of vedolizumab 300 mg or placebo as induction and maintenance therapy for up to 52 weeks. The study designs and outcomes of these trials have been described previously.^16,17^ In this analysis, the vedolizumab study population [*n =* 1434] included all patients who were responders to vedolizumab induction therapy and were subsequently randomised to vedolizumab maintenance therapy [every 4 or 8 weeks] at Week 6, and also those who received induction therapy with vedolizumab, did not achieve clinical response at Week 6, and were then assigned to vedolizumab every 4 weeks for the maintenance phase. The placebo population [*n =* 297] comprised patients who received placebo during the induction phase and were subsequently assigned to continue placebo during the maintenance phase.

To evaluate the incidence of RTI beyond 52 weeks of treatment with vedolizumab, we also included data from the GEMINI long-term safety [LTS] trial, which evaluated the safety of vedolizumab 300 mg administered every 4 weeks in adult patients: ‘rollover’ patients from GEMINI 1 [*n =* 675],^16^ GEMINI 2 [*n =* 726],^17^ GEMINI 3 [*n =* 384],^19^ and a Phase 2 extension study [*n =* 37]^20^; as well as a population of patients [*n =* 421] who were naïve to vedolizumab treatment [*de novo* patients]. Data have previously been reported from the GEMINI LTS trial, also referred to as the long-term safety [LTS] study, for the period May 22, 2009 to June 27, 2013.^21,22^ Follow-up in the study continues and data reported here are from an interim analysis of the data up to May 19, 2015; at this time, 2243 patients had been enrolled [1349 with CD and 894 with UC], constituting 5430 patient-years of follow-up with a median of 981 days’ total exposure to vedolizumab [range 1–2677 days].^23^ Data from the safety population [all patients who received at least one dose of vedolizumab in the GEMINI LTS trial] were analysed. Inclusion and exclusion criteria were as previously reported.^21,22^

### 2.2. Data abstraction and statistical analysis of outcomes

Based on the Medical Dictionary for Regulatory Activities [MedDRA; version 14.0] high-level terms [HLTs] ‘upper respiratory tract infection’ and ‘lower respiratory tract and lung infection’, the incidence of AEs, serious AEs [SAEs], and AEs leading to discontinuation were analysed for each of the GEMINI studies [Supplementary Table 1, available as Supplementary data at *ECCO-JCC* online]. SAEs were defined as those occurring at any dose, regardless of causality, which: were life-threatening or resulted in death; required inpatient hospitalization; resulted in persistent or significant disability/incapacity, congenital anomaly or birth defect; jeopardised the patient and required medical or surgical intervention to prevent any of the above; or involved suspected transmission of an infectious agent. For this analysis, ‘LRTI’ is used as an abbreviation for the MedDRA HLT ‘lower respiratory tract and lung infection’, and ‘events’ refers to AEs. Data for the maintenance phase only were analysed for GEMINI 1 and 2, as well as the LTS trial [including exposure from previous trials]. Exposure-adjusted incidence rates per 100 patient-years were calculated for the incidence of patients with any AE or SAE within the HLTs, as well as for each individual MedDRA preferred term within these. LRTI AEs were analysed by duration of vedolizumab exposure, for the GEMINI LTS trial to evaluate trends over time. The times to first URTI and LRTI event for the GEMINI 1, 2, and LTS trials were also evaluated.

Multivariate logistic regression and Cox proportional hazards modelling were used to identify predictors for the occurrence of any RTI, any URTI, or any LRTI in the individual and pooled GEMINI 1 and GEMINI 2 studies. Predictors assessed were: age; sex; disease duration; previous TNF antagonist use; baseline disease activity; concomitant use of narcotics, corticosteroids, or immunosuppressives; smoking history; and occurrence of surgery during the study. Both unadjusted and adjusted models were evaluated.

## 3. Results

### 3.1. Baseline characteristics

Demographics and baseline characteristics are summarised in Table 1. The mean [standard deviation] exposure to study drug was 228 [132] days for vedolizumab and 154 [119] days for placebo in GEMINI 1 [UC], 203 [136] days for vedolizumab and 172 [129] days for placebo in GEMINI 2 [CD], and 1046 [688] and 915 [656] days for patients with UC and CD, respectively, in GEMINI LTS [including exposure from previous studies].

**Table 1.** Demographics and baseline characteristics in GEMINI 1, GEMINI 2, and GEMINI LTS trials.

Characteristic	GEMINI 1: UC		GEMINI 2: CD		Pooled GEMINI 1 and 2		GEMINI LTS^a^		
	Vedolizumab *n=* 620	Placebo *n=* 149	Vedolizumab *n=* 814	Placebo *n=* 148	Vedolizumab *n=* 1434	Placebo *n=* 297	UC n *=* 894	CD n *=* 1349	Total *n=* 2243
Age, mean [SD] years^b^	40.1 [13.1]	41.2 [12.5]	35.5 [11.9]	38.6 [13.2]	37.5 [12.6]	39.9 [12.9]	41.2 [13.6]	37.8 [12.7]	39.1 [13.2]
Female sex, *n* [%]	256 [41.3]	57 [38.3]	435 [53.4]	79 [53.4]	691 [48.2]	136 [45.8]	372 [41.6]	743 [55.1]	1115 [49.7]
White, *n* [%]	518 [83.5]	115 [77.2]	731 [89.8]	124 [83.8]	1249 [87.1]	239 [80.5]	762 [85.2]	1219 [90.4]	1981 [88]
Body weight, mean [SD] kg	73.4 [18.3]	72.4 [17.7]	70.1 [19.8]	68.7 [18.9]	71.5 [19.2]	70.6 [18.3]	75.2 [18.1]	71.8 [19.3]	73.2 [18.9]
Current smoker, *n* [%]	36 [5.8]	11 [7.4]	216 [26.5]	34 [23.0]	252 [17.6]	45 [15.2]	43 [5.0]^c^	363 [27.1]^d^	406 [18.4]
Former smoker, *n* [%]	204 [32.9]	50 [33.6]	190 [23.3]	29 [19.6]	394 [27.5]	79 [26.6]	272 [31.4]^c^	319 [23.8]^d^	591 [26.8]
Duration of disease, mean [SD] years^e^	6.7 [6.0]	7.1 [7.3]	9.1 [7.5]	8.2 [7.8]	8.0 [7.0]	7.7 [7.5]	8.0 [6.9]	10.1 [8.3]	9.3 [7.9]
Disease duration ≥7 years, *n* [%]	214 [34.5]	53 [35.6]	419 [51.5]	64 [43.2]	633 [44.1]	117 [39.4]	372 [41.6]	755 [56.0]	1127 [50.2]
Baseline disease activity, mean [SD] complete Mayo score	8.6 [1.8]	8.6 [1.7]	−	−	−	−	−	−	−
Baseline disease activity, mean [SD] partial Mayo score	6.0 [1.6]	6.1 [1.5]	−	7	−	−	5.8 [1.8]	−	−
Baseline disease activity, mean [SD] HBI score	–	–	11.2 [3.8]	10.9 [3.7]	−	–	−	10.9 [3.6]	10.9 [3.6]
Baseline disease activity, mean [SD] CDAI score^f^	–	–	323.1 [68.5]	324.6 [78.1]	–	–	−	314.0 [63.2]	314.0 [63.2]
Baseline disease activity score, mean [SD] common index^g^	−	−	−	−	5.8 [1.70]	5.8 [1.70]	−	−	−
In-study surgery, *n* [%]	49 [7.9]	15 [10.1]	126 [15.5]	13 [8.8]	175 [12.2]	28 [9.4]	−	−	−
History of fistulising disease, *n* [%]	–	–	297 [36.5]	56 [37.8]	–	–	–	500 [37.1]	–
Previous TNF antagonist use, *n* [%]	311 [50.2]	73 [49.0]	535 [65.7]	72 [48.6]	846 [59.0]	145 [48.8]	415 [46.4]	898 [66.6]	1313 [58.5]
Any previous TNF antagonist failure, *n* [%]	266 [42.9]	63 [42.3]	497 [61.1]	70 [47.3]	763 [53.2]	133 [44.8]	380 [42.5]	848 [62.9]	1228 [54.7]
Concomitant immunomodulators, *n* [%]	213 [34.4]	44 [29.5]	270 [33.2]	51 [34.5]	483 [33.7]	95 [32.0]	237 [26.5]	381 [28.2]	618 [27.6]
Concomitant corticosteroids, *n* [%]	325 [52.4]	84 [56.4]	417 [51.2]	71 [48.0]	742 [51.7]	155 [52.2]	452 [50.6]	681 [50.5]	1133 [50.5]
Concomitant narcotics, *n* [%]	123 [19.8]	25 [16.8]	276 [33.9]	39 [26.4]	399 [27.8]	64 [21.5]	247 [27.6]	530 [39.3]	777 [34.6]

Previous lung disease was not a specific exclusion criterion. For GEMINI 1 and GEMINI 2, the baseline was Week 0 of each study. Unless otherwise stated, for the GEMINI LTS trial the baseline was the baseline of the previous study for rollover patients and the start of the GEMINI LTS trial for de novo patients.

CD, Crohn’s disease; CDAI, Crohn’s Disease Activity Index; HBI, Harvey–Bradshaw Index; LTS, long-term safety; SD, standard deviation; TNF, tumour necrosis factor; UC, ulcerative colitis.

^a^Interim data cut-off: May 19, 2015.

^b^For the GEMINI LTS trial, age was defined as [1 + first dose date in the GEMINI LTS trial−birth date]/365.25.

^c^ *n =* 865.

^d^ *n =* 1341.

^e^For the GEMINI LTS study data, disease duration defined as [1 + first dose date in the GEMINI LTS trial−diagnosis date]/365.25.

^f^CDAI was not collected for de novo patients.

^g^Baseline disease activity scores based on partial Mayo Score for GEMINI 1 [UC], HBI score for GEMINI 2 [CD], and common index for pooled GEMINI 1 and 2. Common index ranged from 0 to 9 to allow the combination of baseline partial Mayo and HBI scores in the pooled analysis.

### 3.2. Respiratory tract infections in GEMINI 1 and 2

#### 3.2.1. Overall respiratory tract infections

**Figure 1.**
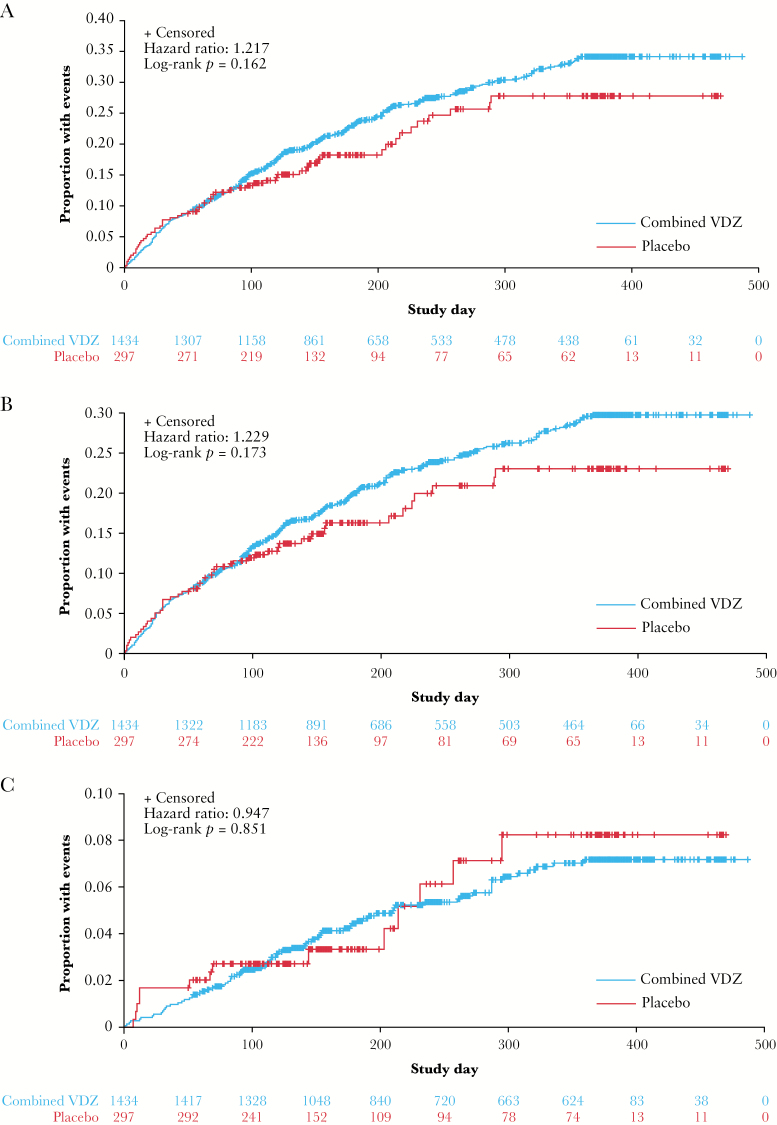
Time to first respiratory tract infection [A], upper respiratory tract infection [B], and lower respiratory tract infection [C]. Kaplan–Meier curves of the time to first respiratory tract infection, upper respiratory tract infection, or lower respiratory tract infection for the placebo and vedolizumab safety populations in the GEMINI 1 and 2 studies combined. Lower respiratory tract infection includes the MedDRA high-level term ‘lower respiratory tract and lung infection’. VDZ, vedolizumab.

No statistically significant differences in time to first lower/upper RTI were observed between patients assigned to vedolizumab therapy and those who received placebo [Figure 1A; HR 1.22, log-rank *p =* 0.162].

#### 3.2.2. Upper respiratory tract infection

No statistically significant differences in time to first URTI were observed between patients assigned to vedolizumab therapy and those who received placebo [Figure 1B; HR 1.23, log-rank *p =* 0.173]. Unadjusted multivariable logistic regression analyses identified previous TNF antagonist use [HR 1.50; 95% CI: 1.20–1.88; *p* < 0.001], smoking [current smokers, HR 1.35; 95% CI: 1.04–1.75; *p =* 0.023], and concomitant narcotic use [HR 1.30; 95% CI: 1.04–1.64; *p =* 0.022] as significant risk factors for the development of a URTI in the pooled GEMINI 1 and 2 population. After adjustment for level of disease activity, these risk factors were still significant: previous TNF antagonist use [HR 1.53; 95% CI: 1.20–1.95; *p =* 0.0007]; smoking [current smokers, HR 1.35; 95% CI: 1.03–1.78; *p =* 0.0316]; and concomitant narcotic use [HR 1.28; 95% CI: 1.00–1.63; *p =* 0.046].

**Figure 2.**
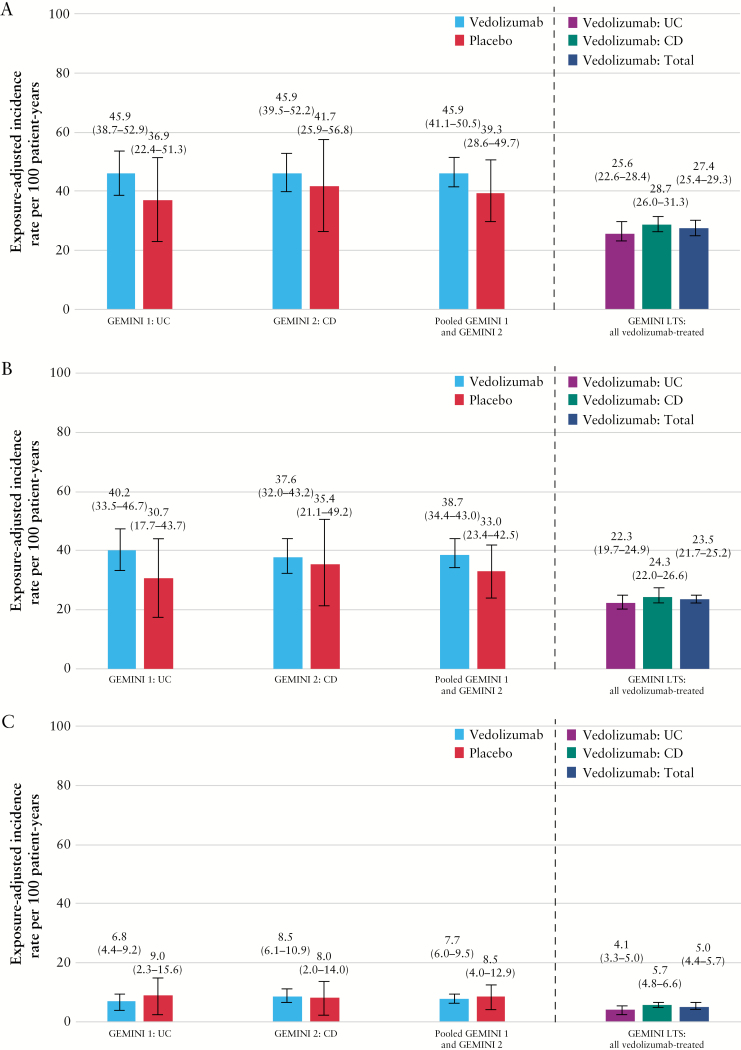
Exposure-adjusted incidence rate per 100 patient-years of upper and lower respiratory tract infections [A], upper respiratory tract infection [B], and lower respiratory tract infection [C] in GEMINI 1, GEMINI 2, and GEMINI LTS trials. Lower respiratory tract infection includes the MedDRA high-level term ‘lower respiratory tract and lung infection’. CD, Crohn’s disease; IR, exposure-adjusted incidence rate per 100 patient-years ([number of patients experiencing an adverse event of interest/total patient exposure time in years] × 100); LTS, long-term safety; UC, ulcerative colitis.

The exposure-adjusted incidence rate of URTIs was numerically lower among patients who received placebo compared with those who received vedolizumab, although the difference was small and not statistically significant [38.7 vs 33.0 patients per 100 patient-years [HR 1.12; 95% CI: 0.83–1.51; *p =* 0.463]; Figure 2 and Supplementary Table 2, available as Supplementary data at *ECCO-JCC* online]. Two patients who received vedolizumab had a serious URTI and one patient discontinued treatment due to laryngitis [Supplementary Table 2]. No patients receiving placebo had a serious URTI or discontinued therapy due to a URTI [Supplementary Table 2]. No deaths from URTIs occurred in either group.

The most frequently reported URTI, nasopharyngitis, occurred at a higher rate in the vedolizumab group than in patients who received placebo [18.6 vs 12.8 patients per 100 patient-years in the pooled GEMINI 1 and 2 populations; Table 2]. URTI was the next most common URTI [MedDRA preferred term] and the incidence rate was similar for vedolizumab-treated and placebo-receiving patients [10.5 vs 11.6 patients per 100 patient-years].

**Table 2.** Exposure-adjusted incidence rate per 100 patient-years of respiratory tract infections in the GEMINI 1, GEMINI 2, and GEMINI LTS trials by preferred terms.

Category	GEMINI 1: UC				GEMINI 2: CD				Pooled GEMINI 1 and GEMINI 2				GEMINI LTS: all vedolizumab-treated patients^a^					
Safety population	Vedolizumab [*n=* 620] TPY *=* 471.9		Placebo [*n=* 149] TPY *=* 81.2		Vedolizumab [*n=* 814] TPY *=* 586.2		Placebo[*n=* 148TPY *=* 90.1		Vedolizumab [*n=* 1434] TPY *=* 1058.2		Placebo [*n=* 297] TPY *=* 171.3		UC [*n=* 894] TPY *=* 2285.4		CD [*n=* 1349] TPY *=* 3145.0		Total [*N=* 2243] TPY *=* 5430.3	
	*n* [%]	IR^b^ [95% CI]	*n* [%]	IR^b^ [95% CI]	*n* [%]	IR^b^ [95% CI]	*n* [%]	IR^b^ [95% CI]	*n* [%]	IR^b^ [95% CI]	*n* [%]	IR^b^ [95% CI]	*n* [%]	IR^b^ [95% CI]	*n* [%]	IR^b^ [95% CI]	*n* [%]	IR^b^ [95% CI]
Upper respiratory tract infections																		
URTIs	155 [25.0]	40.1 [33.5–46.7]	23 [15.4]	30.7 [17.7–43.7]	184 [22.6]	37.6 [32.0–43.2]	27 [18.2]	35.2 [21.1–49.2]	339 [23.6]	38.7 [34.4–43.0]	50 [16.8]	33.0 [23.4–42.6]	347[38.8]	22.3 [19.7–24.9]	518 [38.4]	24.3 [22.0–26.7]	865 [38.6]	23.5 [21.7–25.2]
Nasopharyngitis	80 [12.9]	18.6 [14.5–22.8]	11 [7.4]	14.0 [5.5–22.4]	100 [12.3]	18.7 [14.9–22.4]	10 [6.8]	11.8 [4.3–19.3]	180 [12.6]	18.6 [15.9–21.4]	21 [7.1]	12.8 [7.2–18.5]	203 [22.7]	10.8 [9.3–12.4]	285 [21.1]	11.0 [9.6–12.3]	488 [21.8]	10.9 [9.9–11.9]
URTI [preferred term]	52 [8.4]	11.7 [8.5–14.9]	8 [5.4]	10.1 [3.1–17.1]	54 [6.6]	9.6 [7.0–12.2]	11 [7.4]	13.0 [5.1–20.8]	106 [7.4]	10.5 [8.5–12.6]	19 [6.4]	11.6 [6.3–16.9]	130 [14.5]	6.4 [5.3–7.5]	172 [12.8]	6.0 [5.0–6.9]	302 [13.5]	6.1 [5.4–6.8]
Sinusitis	15 [2.4]	3.2 [1.6–4.9]	2 [1.3]	2.5 [0.0–6.0]	29 [3.6]	5.1 [3.2–6.9]	1 [0.7]	1.1 [0.0–3.3]	44 [3.1]	4.3 3.0–5.5]	3 [1.0]	1.8 [0.0–3.8]	75 [8.4]	3.5 [2.7–4.3]	121 [9.0]	4.1 [3.4–4.9]	196 [8.7]	3.8 [3.3–4.4]
Pharyngitis	9 [1.5]	1.9 [0.7–3.2]	0	–	15 [1.8]	2.6 [1.3–3.9]	1 [0.7]	1.1 [0.0–3.3]	24 [1.7]	2.3 [1.4–3.2]	1 [0.3]	0.6 [0.0–1.7]	28 [3.1]	1.2 [0.8–1.7]	38 [2.8]	1.2 [0.8–1.6]	66 [2.9]	1.2 [0.9–1.5]
Rhinitis	8 [1.3]	1.7 [0.5–2.9]	3 [2.0]	3.8 [0.0–8.1]	5 [0.6]	0.9 [0.1–1.6]	1 [0.7]	1.1 [0.0–3.3]	13 [0.9]	1.2 [0.6–1.9]	4 [1.3]	2.4 [<0.1–4.7]	10 [1.1]	0.4 [0.1–0.7]	25 [1.9]	0.8 [0.5–1.1]	35 [1.6]	0.6 [0.4–0.8]
Tonsillitis	4 [0.6]	0.9 [<0.1–1.7]	0	–	1 [0.1]	0.2 [0.0–0.5]	0	–	5 [0.3]	0.5 [0.1–0.9]	0	–	10 [1.1]	0.4 [0.2–0.7]	15[1.1]	0.5 [0.2–0.7]	25 [1.1]	0.5 [0.3–0.6]
Laryngitis	2 [0.3]	0.4 [0.0–1.0]	0	–	1 [0.1]	0.2 [0.0–0.5]	2 [1.4]	2.2 [0.0–5.3]	3 [0.2]	0.3 [0.0–0.6]	2 [0.7]	1.2 [0.0–2.8]	3 [0.3]	0.1 [0.0–0.3]	16 [1.2]	0.5 [0.3–0.8]	19 [0.8]	0.4 [0.2–0.5]
Tracheitis	1 [0.2]	0.2 [0.0–0.6]	0	–	2 [0.2]	0.3 [0.0–0.8]	1 [0.7]	1.1 [0.0–3.3]	3 [0.2]	0.3 [0.0–0.6]	1 [0.3]	0.6 [0.0–1.7]	2 [0.2]	0.1 [0.0–0.2]	0	–	2 [<0.1]	<0.1 [0.0–0.1]
Acute sinusitis	0	–	1 [0.7]	1.2 [0.0–3.7]	2 [0.2]	0.3 [0.0–0.8]	0	–	2 [0.1]	0.2 [0.0–0.5]	1 [0.3]	0.6 [0.0–1.7]	3 [0.3]	0.1 [0.0–0.3]	5 [0.4]	0.2 [<0.1–0.3]	8 [0.4]	0.2 [<0.1–0.3]
Acute tonsillitis	1 [0.2]	0.2 [0.0–0.6]	0	–	1 [0.1]	0.2 [0.0–0.5]	1 [0.7]	1.1 [0.0–3.3]	2 [0.1]	0.2 [0.0–0.5]	1 [0.3]	0.6 [0.0–1.7]	5 [0.6]	0.2 [<0.1–0.4]	11 [0.8]	0.4 [0.1–0.6]	16 [0.7]	0.3 [0.2–0.4]
Chronic sinusitis	1 [0.2]	0.2 [0.0–0.6]	0	–	1 [0.1]	0.2 [0.0–0.5]	0	–	2 [0.1]	0.2 [0.0–0.5]	0	–	4 [0.4]	0.1 [0.0–0.3]	4 [0.3]	0.1 [<0.1–0.3]	8 [0.4]	0.1 [<0.1–0.2]
Sinobronchitis	0	–	0	–	0	–	0	–	0	–	0	–	1 [0.1]	<0.1 [0.0–0.1]	1 [<0.1]	<0.1 [0.0–0.1]	2 [<0.1]	<0.1 [0.0–0.1]
Tracheobronchitis	1 [0.2]	0.2 [0.0–0.6]	0	–	0	–	0	–	1 [<0.1]	0.1 [0.0–0.3]	0	–	0	–	1 [<0.1]	<0.1	1 [<0.1]	<0.1 [0.0–0.1]
Rhinolaryngitis	0	–	0	–	0	–	1 [0.7]	1.1 [0.0–3.3]	0	–	1 [0.3]	0.6 [0.0–1.7]	0	–	0	–	0	–
Peritonsillar abscess	0	–	0	–	0	–	0	–	0	–	0	–	1 [0.1]	<0.1 [0.0–0.1]	0	–	1 [<0.1]	<0.1 [0.0–0.1]
Pharyngotonsillitis	0	–	0	–	0	–	0	–	0	–	0	–	1 [0.1]	<0.1 [0.0–0.1]	0	–	1 [<0.1]	<0.1 [0.0–0.1]
Serious URTIs	1 [0.2]	0.2 [0.0–0.6]	0	–	1 [0.1]	0.2 [0.0–0.5]	0	–	2 [0.1]	0.2 [0.0–0.5]	0	–	2 [0.2]	0.1 [0.0–0.2]	4 [0.3]	0.1 [<0.1–0.3]	6 [0.3]	0.1 [<0.1–0.2]
Acute sinusitis	0	–	0	–	1 [0.1]	0.2 [0.0–0.5]	0	–	1 [<0.1]	0.1 0.0–0.3]	0	–	0	–	0	–	0	–
Sinusitis	0	–	0	–	1 [0.1]	0.2 [0.0–0.5]	0	–	1 [<0.1]	0.1 [0.0–0.3]	0	–	0	–	2 [0.1]	0.1 [0.0–0.2]	2 [<0.1]	<0.1 [0.0–0.1]
Tonsillitis	1 [0.2]	0.2 [0.0–0.6]	0	–	0	–	0	–	1 [<0.1]	0.1 [0.0–0.3]	0	–	0	–	0	–	0	–
URTI [preferred term]	0	–	0	–	0	–	0	–	0	–	0	–	1 [0.1]	<0.1 [0.0–0.1]	0	–	1 [<0.1]	<0.1 [0.0–0.1]
Laryngitis	0	–	–	–	0	–	0	–	0	–	0	–	0	–	1 [<0.1]	<0.1 [0.0–0.1]	1 [<0.1]	<0.1 [0.0–0.1]
Nasopharyngitis	0	–	–	–	–	–	0	–	0	–	0	–	0	–	1 [<0.1]	<0.1 [0.0–0.1]	1 [<0.1]	<0.1 [0.0–0.1]
Peritonsillar abscess	0	–	–	–		–	0	–	0	–	0	–	1 [0.1]	<0.1 [0.0–0.1]	0	–	1 [<0.1]	<0.1 [0.0–0.1]
Lower respiratory tract infections																		
LRTIs	31 [5.0]	6.7 [4.4–9.2]	7 [4.7]	9.0 [2.3–15.6]	48 [5.9]	8.5 [6.1–10.9]	7 [4.7]	8.0 [2.0–14.0]	79 [5.5]	7.7 [6.0–9.4]	14 [4.7]	8.5 [4.0–12.9]	88 [9.8]	4.1 [3.2–5.0]	163 [12.1]	5.7 [4.8–6.5]	251 [11.2]	5.0 [4.4–5.6]
Bronchitis	24 [3.9]	5.2 [3.1–7.3]	5 [3.4]	6.4 [0.7–12.0]	33 [41]	5.8 3.8–7.8]	5 [3.4]	5.7 [0.6–10.8]	57 [4.0]	5.5 [4.1–7.0]	10 [3.4]	6.0 [2.2–9.8]	54 [6.0]	2.4 [1.8–3.1]	118 [8.7]	4.0 [3.3–4.7]	172 [7.7]	3.3 [2.8–3.8]
Pneumonia	2 [0.3]	0.4 [0.0–1.0]	1 [0.7]	1.2 [0.0–3.7]	9 [1.1]	1.5 [0.5–2.6]	1 [0.7]	1.1 [0.0–3.3]	11 [0.8]	1.0 [0.4–1.7]	2 [0.7]	1.2 [0.0–2.8]	20 [2.2]	0.9 [0.5–1.3]	29 [2.1]	0.9 [0.6–1.3]	49 [2.2]	0.9 [0.7–1.2]
LRTI [preferred term]	5 [0.8]	1.1 [0.1–2.0]	1 [0.7]	1.2 [0.0–3.6]	5 [0.6]	0.9 [0.1–1.6]	0	–	10 [0.7]	1.0 [0.4–1.5]	1 [0.3]	0.6 [0.0–1.7]	17 [1.9]	0.8 [0.4–1.1]	29 [2.1]	0.9 [0.6–1.2]	46 [2.1]	0.8 [0.6–1.1]
Bronchopneumonia	1 [0.2]	0.2 [0.0–0.6]	0	–	0	–	1 [0.7]	1.1 [0.0–3.3]	1 [0.1]	0.1 [0.0–0.3]	1 [0.3]	0.6 [0.0–1.7]	0	–	1 [<0.1]	<0.1 [0.0–0.1]	1 [<0.1]	<0.1 [0.0–0.1]
Lung infection	0	–	0	–	1 [0.1]	0.2 [0.0–0.5]	0	–	1 [<0.1]	0.1 [0.0–0.3]	0	–	0	–	1 [<0.1]	<0.1 [0.0–0.1]	1 [<0.1]	<0.1 [0.0–0.1]
Primary atypical pneumonia	0	–	0	–	1 [0.1]	0.2 [0.0–0.5]	0	–	1 [0.1]	0.1 [0.0–0.3]	0	–	0	–	1 [<0.1]	<0.1 [0.0–0.1]	1 [<0.1]	<0.1 [0.0–0.1]
Lobar pneumonia	0	–	0	–	0	–	0	–	0	–	0	–	2 [0.2]	0.1 [0.0–0.2]	3 [0.2]	0.1 [0.0–0.2]	5 [0.2]	0.1 [<0.1–0.2]
Serious LRTIs	2 [0.3]	0.4 [0.0–1.0]	0	–	3 [0.4]	0.5 [0.0–1.1]	1 [0.7]	1.1 [0.0–3.3]	5 [0.3]	0.5 [0.1–0.9]	1 [0.3]	0.6 [0.0–1.7]	10 [1.1]	0.4 [0.2–0.7]	11 [0.8]	0.3 [0.1–0.5]	21 [0.9]	0.4 [0.2– 0.5]
Pneumonia	0	–	0	–	2 [0.2]	0.3 [0.0–0.8]	0	–	2 [0.1]	0.2 [0.0–0.5]	0	–	8 [0.9]	0.4 [0.1–0.6]	11 [0.8]	0.3 [0.1–0.5]	19 [0.8]	0.3 [0.2–0.5]
Bronchitis	1 [0.2]	0.2 [0.0–0.6]	0	–	0	–	0	–	1 [<0.1]	0.1 [0.0–0.3]	0	–	0	–	0	–	0	–
LRTI [preferred term]	1 [0.2]	0.2 [0.0–0.6]	0	–	0	–	0	–	1 [<0.1]	0.1 [0.0–0.3]	0	–	0	–	0	–	0	–
Lobar pneumonia	0	–	0	–	0	–	0	–	0	–	0	–	2 [0.2]	0.1 [0.0–0.2]	0	–	2 [<0.1]	<0.1 [0.0–0.1]
Lung infection	0	–	0	–	1 [0.1]	0.2 [0.0–0.5]	0	–	1 [<0.1]	0.1 [0.0–0.3]	0	–	0	–	0	–	0	–
Bronchopneumonia	0	–	0	–	0	–	1 [0.7]	1.1 [0.0–3.3]	0	–	1 [0.3]	0.6 [0.0–1.7]	0	–	0	–	0	–

Except where otherwise noted, LRTI is defined according to the MedDRA high-level term ‘lower respiratory tract and lung infection’. Patients with one or more adverse events within a level of the MedDRA term are counted only once in that level.

CD, Crohn’s disease; CI, confidence interval; IR, incidence rate; LRTI, lower respiratory tract infection; MedDRA, Medical Dictionary for Regulatory Activities; LTS, long-term safety; TPY, total number of patient-years of exposure; UC, ulcerative colitis; URTI, upper respiratory tract infection.

^a^Interim data cut-off: May 19, 2015.

^b^IR, exposure-adjusted incidence rate per 100 patient-years ([number of patients experiencing an adverse event of interest/total patient exposure time in years] × 100).

#### 3.3.3. Lower respiratory tract infection

No statistically significant differences in time to first LRTI were observed between patients assigned to vedolizumab therapy and those who received placebo [Figure 1C; HR 0.95, log-rank *p =* 0.851]. In unadjusted multivariable logistic regression analyses, previous TNF antagonist use [HR 2.20; 95% CI: 1.10–4.41; *p =* 0.027] and female sex [HR 2.11; 95% CI: 1.07–4.14; *p =* 0.030] were associated with LRTI in patients with UC, whereas in the CD population, current smoker status alone was associated with the occurrence of LRTI [HR 2.37; 95% CI: 1.26–4.45; *p =* 0.008; Table 3]. A higher proportion of patients with CD were current smokers compared with those with UC [29.2% of females and 22.3% of males in GEMINI 2; 4.2% of females and 7.5% of males in GEMINI 1]. Vedolizumab therapy, age, disease duration, baseline disease activity, or concomitant narcotic, corticosteroid, and immunosuppressive use were not associated with a significantly greater risk of LRTI. After adjustment for disease activity, none of the evaluated risk factors were significant predictors of LRTI in patients with UC [Table 4]. However, smoking [current smokers, HR 3.43; 95% CI: 1.67–7.04; *p =* 0.0008] remained a significant predictor of LRTI in patients with CD.

**Table 3.** Selected respiratory tract infection treatment emergent adverse events in the pooled GEMINI 1 and GEMINI 2 studies by preferred terms, time-adjusted rates [safety population].

	Vedolizumab Q8 Wks [*n=* 276] [TPY *=* 220.7]				Vedolizumab Q4 Wks [*n=* 1158] [TPY *=* 837.5]			
	Total no. of subjects	Time-adjusted patient-years	Incidence rate	95% CI	Total no. of subjects	Time-adjusted patient-years	Incidence rate	95% CI
Upper respiratory tract infections								
URTI TEAEs	70	184.3	37.98	[28.75–47.21]	269	691.9	38.88	[34.05–43.71]
Nasopharyngitis	42	201.4	20.8	[14.41–27.30]	138	764.1	18.06	[15.00–21.12]
Upper respiratory tract infection	19	210.1	9.04	[4.923–13.164]	87	795.5	10.94	[8.610–13.261]
Sinusitis	7	216.9	3.23	[0.830–5.625]	37	817.8	4.52	[3.063–5.986]
Pharyngitis	4	218.7	1.83	[0.040–3.618]	20	826.7	2.42	[1.357–3.482]
Rhinitis	2	219.8	0.91	[0.000–2.170]	11	831.5	1.32	[0.539–2.107]
Tonsillitis	0	220.7	0.00	[0.000–0.000]	5	835.6	0.60	[0.074–1.123]
Laryngitis	0	220.7	0.00	[0.000–0.000]	3	835.4	0.36	[0.000–0.766]
Tracheitis	0	220.7	0.00	[0.000–0.000]	3	835.3	0.36	[0.000–0.766]
Acute tonsillitis	0	220.7	0.00	[0.000–0.000]	2	836.2	0.24	[0.000–0.571]
Chronic sinusitis	0	220.7	0.00	[0.000–0.000]	2	836.4	0.24	[0.000–0.571]
Acute sinusitis	1	220.0	0.45	[0.000–1.346]	1	836.7	0.12	[0.000–0.354]
Tracheobronchitis	1	219.7	0.46	[0.000–1.349]	0	837.5	0.00	[0.000–0.000]
URTI SAEs	0	220.7	0.00	[0.000–0.000]	2	836.6	0.24	[0.000–0.570]
Acute sinusitis	0	220.7	0.00	[0.000–0.000]	1	836.7	0.12	[0.000–0.354]
Sinusitis	0	220.7	0.00	[0.000–0.000]	1	836.7	0.12	[0.000–0.354]
Tonsillitis	0	220.7	0.00	[0.000–0.000]	1	837.4	0.12	[0.000–0.353]
Lower respiratory tract infections								
LRTI TEAEs	14	215.0	6.51	[3.057–9.965]	65	806.8	8.06	[6.091–10.023]
Bronchitis	12	215.6	5.57	[2.381–8.751]	45	814.8	5.52	[3.902–7.144]
Lower respiratory tract infection	1	220.3	0.45	[0.000–1.344]	9	832.8	1.08	[0.375–1.786]
Pneumonia	2	220.1	0.91	[0.000–2.172]	9	834.5	1.08	[0.374–1.783]
Bronchopneumonia	0	220.7	0.00	[0.000–0.000]	1	837.1	0.12	[0.000–0.354]
Lung infection	0	220.7	0.00	[0.000–0.000]	1	837.4	0.12	[0.000–0.354]
Pneumonia primary atypical	0	220.7	0.00	[0.000–0.000]	1	837.2	0.12	[0.000–0.354]
LRTI SAEs	2	219.4	0.91	[0.000–2.176]	3	837.0	0.36	[0.000–0.764]
Pneumonia	0	220.7	0.00	[0.000–0.000]	2	837.1	0.24	[0.000–0.570]
Lung infection	0	220.7	0.00	[0.000–0.000]	1	837.4	0.12	[0.000–0.354]
Bronchitis	1	219.8	0.45	[0.000–1.348]	0	837.5	0.00	[0.000–0.000]
Lower respiratory tract infection	1	220.3	0.45	[0.000–1.344]	0	837.5	0.00	[0.000–0.000]

CI, confidence interval; LRTI, lower respiratory tract infection; SAE, serious adverse event; TEAE, treatment emergent adverse event; TPY, total number of patient-years of exposure; URTI, upper respiratory tract infection, WKs, weeks.

**Table 4.** Predictors of lower respiratory tract infections in GEMINI 1 and 2.^a^

Variable	All patients	Patients with AE	Adjusted results	
			HR [95% CI]	*p*-Value
**UC population [GEMINI 1]**	***n*** *=* **769**	***n*** *=* **38**		
Age, mean [SD] years	40.3 [13.0]	42.5 [13.3]	1.00 [0.98–1.03]	0.770
Female sex, *n* [%]	313 [40.7]	22 [57.9]	2.11 [1.07–4.14]	0.030
Disease duration ≥7 years, *n* [%]	267 [34.7]	18 [47.4]	1.49 [0.77–2.88]	0.242
Previous TNF antagonist therapy, *n* [%]	384 [49.9]	25 [65.8]	2.20 [1.10–4.41]	0.027
Baseline disease activity, mean [SD] complete Mayo score	6.1 [1.6]	5.8 [1.6]	0.97 [0.79–1.18]	0.733
Concomitant narcotic use, *n* [%]	148 [19.2]	8.0 [21.1]	0.82 [0.37–1.83]	0.628
Concomitant corticosteroid use, *n* [%]	409 [53.2]	19 [50.0]	0.76 [0.40–1.46]	0.415
Concomitant immunomodulator use, *n* [%]	257 [33.4]	16 [42.1]	1.51 [0.79–2.91]	0.217
Current smoker, *n* [%]^b^	47 [6.1]	1.0 [2.6]	0.61 [0.08–4.63]	0.635
Former smoker, *n* [%]^b^	254 [33.0]	18 [47.4]	2.01 [0.99–4.07]	0.053
In-study surgery, *n* [%]	64 [8.3]	4.0 [10.5]	1.11 [0.25–4.92]	0.896
Vedolizumab treatment, *n* [%]	620 [80.6]	31 [81.6]	0.72 [0.31–1.69]	0.452
**CD population [GEMINI 2]**	***n*** *=* **962**	***n*** *=* **55**		
Age, mean [SD] years	35.9 [12.14]	37.1 [12.19]	0.99 [0.97–1.02]	0.603
Female sex, *n* [%]	514 [53.4]	37 [67.3]	1.64 [0.92–2.92]	0.093
Disease duration ≥7 years, *n* [%]	483 [50.2]	34 [61.8]	1.35 [0.75–2.43]	0.324
Previous TNF antagonist therapy, *n* [%]	607 [63.1]	40 [72.7]	1.41 [0.73–2.72]	0.303
Baseline disease activity, mean [SD] score	11.2 [3.8]	12.0 [3.7]	1.05 [0.98–1.13]	0.136
Concomitant narcotic use, *n* [%]	315 [32.7]	20 [36.4]	0.85 [0.47–1.54]	0.597
Concomitant corticosteroid use, *n* [%]	488 [50.7]	24 [43.6]	0.83 [0.49–1.42]	0.493
Concomitant immunomodulator use, *n* [%]	321 [33.4]	13 [23.6]	0.67 [0.36–1.27]	0.225
Current smoker, *n* [%]^b^	250 [26.0]	24 [43.6]	2.37 [1.26–4.45]	0.008
Former smoker, *n* [%]^b^	219 [22.8]	13 [23.6]	1.51 [0.73–3.15]	0.271
In-study surgery, *n* [%]	139 [14.4]	10 [18.2]	2.03 [0.83–4.95]	0.119
Vedolizumab treatment, *n* [%]	814 [84.6]	48 [87.3]	0.94 [0.42–2.10]	0.872
**Combined UC and CD population [GEMINI 1 and 2]**	***n*** *=* **1731**	***n*** *=* **93**		
Age, mean [SD] years	37.9 [12.7]	39.3 [12.8]	1.00 [0.98–1.02]	0.994
Female sex, *n* [%]	827 [47.8]	59 [63.4]	1.84 [1.19–2.83]	0.006
Disease duration ≥7 years, *n* [%]	750 [43.3]	52 [55.9]	1.35 [0.88–2.09]	0.173
Previous TNF antagonist therapy, *n* [%]	991 [57.3]	65 [69.9]	1.70 [1.06–2.73]	0.028
Baseline disease activity, mean [SD] score^c^	5.8 [1.7]	5.9 [1.7]	1.07 [0.95–1.22]	0.278
Concomitant narcotic use, *n* [%]	463 [26.7]	28 [30.1]	0.87 [0.55–1.39]	0.565
Concomitant corticosteroid use, *n* [%]	897 [51.8]	43 [46.2]	0.83 [0.55–1.25]	0.382
Concomitant immunomodulator use, *n* [%]	578 [33.4]	29 [31.2]	0.96 [0.62–1.50]	0.862
Current smoker, *n* [%]^b^	297 [17.2]	25 [26.9]	1.97 [1.17–3.31]	0.011
Former smoker, *n* [%]^b^	473 [27.3]	31 [33.3]	1.74 [1.06–2.87]	0.029
In-study surgery, *n* [%]	203 [11.7]	14 [15.1]	1.65 [0.78–3.49]	0.195
Vedolizumab treatment, *n* [%]	1434 [82.8]	79 [84.9]	0.85 [0.48–1.52]	0.585

LRTI is defined according to the MedDRA high-level term ‘lower respiratory tract and lung infection’.

AE, adverse event; CD, Crohn’s disease; CI, confidence interval; HBI, Harvey–Bradshaw Index; HR, hazard ratio, SD, standard deviation; TNF, tumour necrosis factor; UC, ulcerative colitis.

^a^Analysis based on Cox proportional hazards model.

^b^Current and former smoker estimates are relative to non-smokers.

^c^Baseline disease activity scores based on partial Mayo Score for GEMINI 1 [UC], HBI score for GEMINI 2 [CD], and common index for pooled GEMINI 1 and 2. Common index ranged from 0 to 9 to allow the combination of baseline partial Mayo and HBI scores in the pooled analysis.

The exposure-adjusted incidence rate of LRTIs was numerically lower for vedolizumab-treated patients than for placebo recipients in the pooled GEMINI 1 and GEMINI 2 analysis, although this difference was not statistically significant (7.7 vs 8.5 patients per 100 patient-years [HR 0.85; 95% CI: 0.48–1.52; *p =* 0.585]; Figure 2 and Supplementary Table 2). The rate [95% CI] of serious LRTIs was low in both vedolizumab and placebo groups (0.5 [0.1–0.9] and 0.6 [0.0–1.7] patients per 100 patient-years, respectively; Supplementary Table 2). There were two LRTI-related discontinuations in the vedolizumab group [bronchitis and pneumonia, both in patients with CD] compared with none in the placebo group [Supplementary Table 2]. There was one LRTI-related death, which occurred in a patient receiving placebo who died from bronchopneumonia. Patients with UC who received treatment with vedolizumab had a statistically non-significant lower rate of LRTIs compared with those receiving placebo (6.8 vs 9.0 per 100 patient-years [HR 0.72; 95% CI: 0.31–1.69; *p =* 0.452]; Figure 2 and Table 3). For patients with CD, the corresponding rates were 8.5 and 8.0 per 100 patient-years for the vedolizumab and placebo groups, respectively [HR 0.94; 95% CI: 0.42–2.10; *p =* 0. 872; Table 3]. Bronchitis was the most frequently reported LRTI in both the vedolizumab and the placebo groups in the GEMINI 1 and 2 populations. The rate was similar for the vedolizumab and placebo groups in the pooled GEMINI 1 and 2 data analysis (5.5 vs 6.0 patients per 100 patient-years [HR 0.84; 95% CI: 0.42–1.66; *p =* 0.607]; Table 2). Overall the incidence rates [95% CI] of pneumonia, bronchopneumonia, and primary atypical pneumonia were similar with vedolizumab therapy compared with placebo (1.0 [0.4–1.7] vs 1.2 [0.0–2.8], 0.1 [0.0–0.3] vs 0.6 [0.0–1.7], and 0.1 [0.0–0.3] vs 0 patients per 100 patient-years, respectively; Table 2). Two of these events in the vedolizumab group were serious, both in patients with CD. The fatal event of bronchopneumonia in a patient with CD was the only serious event in the placebo group.

#### 3.3.4. Respiratory tract infections based on frequency of vedolizumab maintenance therapy

**Figure 3.**
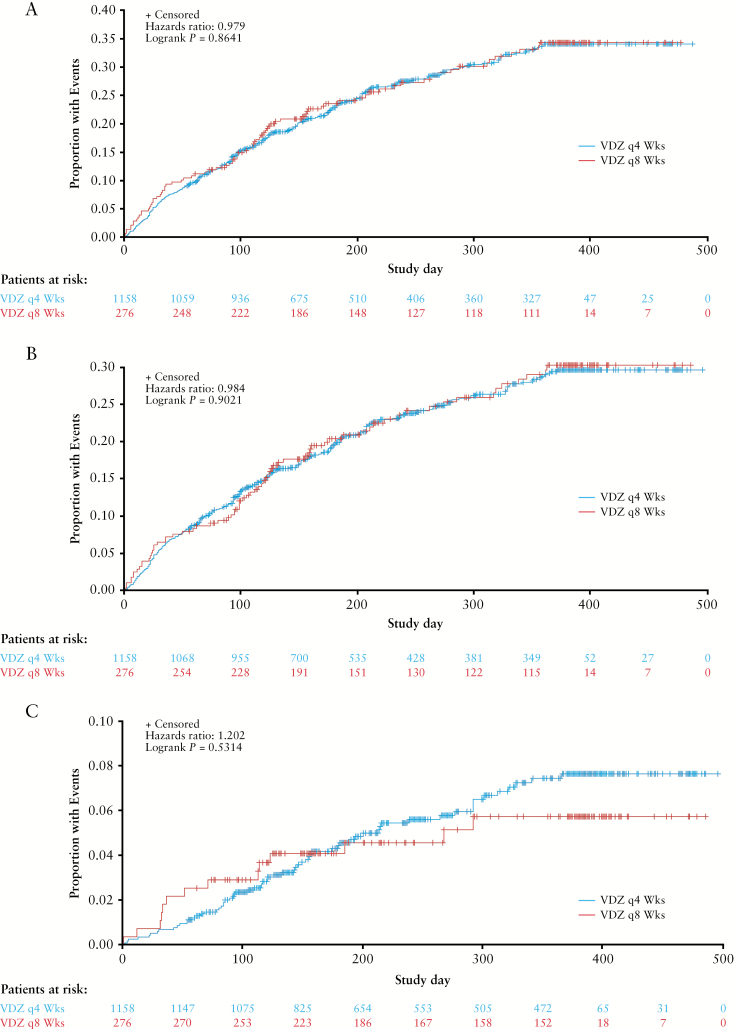
Time to first respiratory tract infection [A], upper respiratory tract infection [B], and lower respiratory tract infection [C]. Kaplan–Meier curves of the time to first respiratory tract infection, upper respiratory tract infection, or lower respiratory tract infection for the combined GEMINI 1 and 2 study vedolizumab 4-week and 8-week maintenance therapy safety populations. Lower respiratory tract infection includes the MedDRA high-level term ‘lower respiratory tract and lung infection’. VDZ, vedolizumab.

**Figure 4.**
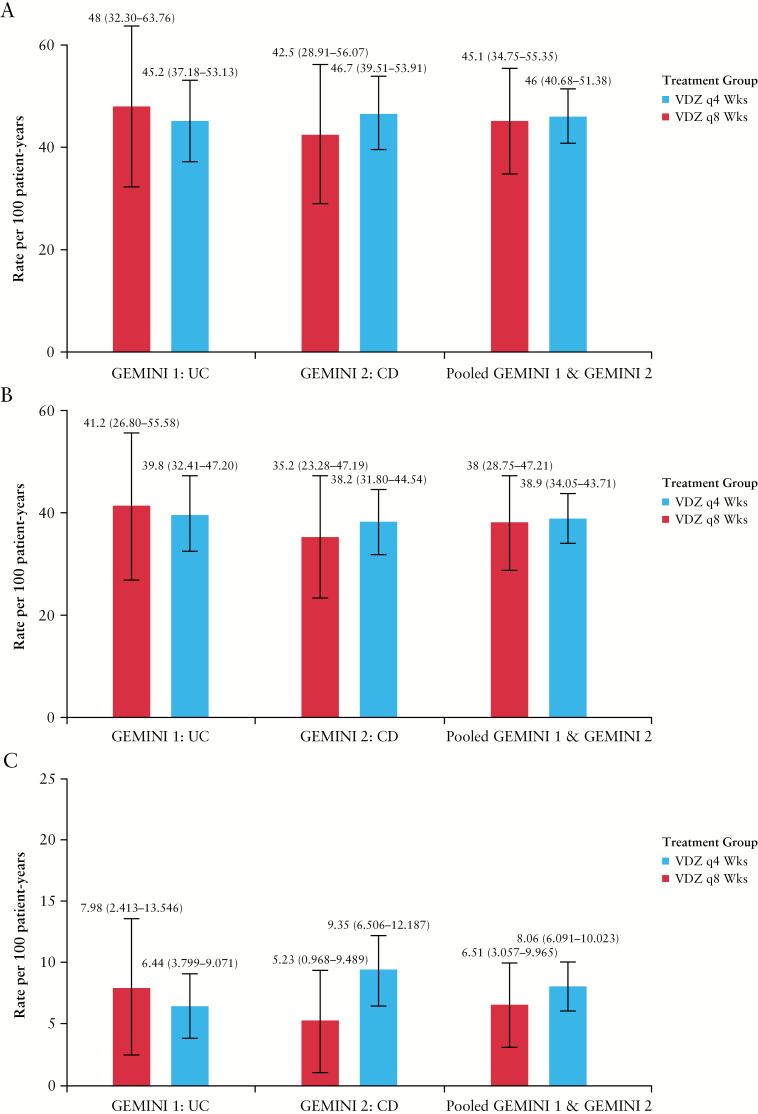
Exposure-adjusted incidence rate per 100 patient-years of respiratory tract infections [A], upper respiratory tract infection [B], and lower respiratory tract infection [C] in GEMINI 1, GEMINI 2, and combined GEMINI 1 and GEMINI 2 safety populations. Patients received vedolizumab every 4 weeks or every 8 weeks as maintenance therapy. Lower respiratory tract infection includes the MedDRA high-level term ‘lower respiratory tract and lung infection’. CD, Crohn’s disease; IR, exposure-adjusted incidence rate per 100 patient-years ([number of patients experiencing an adverse event of interest/total patient exposure time in years] × 100); LTS, long-term safety; UC, ulcerative colitis.

No statistically significant differences were observed between patients assigned to vedolizumab maintenance therapy every 4 weeks vs every 8 weeks in time to first RTI [Figure 3A; HR 0.98, log-rank *p =* 0.864], time to first URTI [Figure 3B; HR 0.98, log-rank *p =* 0.902] or time to first LRTI [Figure 3C; HR 1.20, log-rank *p =* 0.531]. Similarly, no significant between-group differences were found in exposure-adjusted incidence rates of RTI, URTI, or LRTI [Figure 4].

Two patients who received vedolizumab maintenance therapy every 4 weeks had a serious URTI and one patient discontinued treatment due to a serious URTI [Supplementary Table 3, available as Supplementary data at *ECCO-JCC* online]. No patients receiving vedolizumab maintenance therapy every 8 weeks had a serious URTI and no patients discontinued therapy due to a serious URTI [Supplementary Table 3]. Three patients who received vedolizumab maintenance therapy every 4 weeks had a serious LRTI and one patient discontinued treatment due to serious LRTI [Supplementary Table 3]. Finally, two patients receiving vedolizumab maintenance therapy every 8 weeks had a serious LRTI and no patients discontinued therapy due to a serious LRTI [Supplementary Table 3].

The most frequently reported URTI, nasopharyngitis, occurred at a slightly lower rate in the vedolizumab 4-week group than in the vedolizumab 8-week group [18.1 vs 20.8 patients per 100 patient-years in the pooled GEMINI 1 and 2 populations; Table 3]. Upper respiratory tract infection was the next most common URTI [MedDRA preferred term] and the incidence rate was similar in the vedolizumab 4-week and vedolizumab 8-week treatment groups [10.9 vs 9.0 patients per 100 patient-years]. Bronchitis was the most frequently reported LRTI in both the vedolizumab 4-week and vedolizumab 8-week treatment groups in the GEMINI 1 and 2 studies. The rate of bronchitis was similar for both groups in the pooled GEMINI 1 and 2 data analysis [5.52 vs 5.57 patients per 100 patient-years; Table 3]. Overall the incidence rates [95% CIs] of pneumonia, bronchopneumonia, and primary atypical pneumonia were similar with vedolizumab 4-week maintenance therapy compared with vedolizumab 8-week maintenance therapy (1.1 [0.4─1.8] vs 0.9 [0─2.2], 0.1 [0.0–0.4] vs 0, and 0.1 [0─0.4] vs 0 patients per 100 patient-years, respectively; Table 3). Two of the pneumonia events in the vedolizumab 4-week group and one of the bronchitis events in the vedolizumab 8-week group were serious.

### 3.4. Respiratory tract infections in the open-label extension

In the GEMINI LTS trial, exposure-adjusted incidence rates for URTI [95% CI] were numerically lower for vedolizumab-treated patients with UC than for those with CD (22.3 [19.7–24.9] vs 24.3 [22.0–26.7] events per 100 patient-years; Figure 2 and Supplementary Table 2). Nasopharyngitis and URTI [MedDRA preferred term] were the most frequent URTI events [Table 2].

There was no increase in the incidence rates [95% CI] of LRTIs in the GEMINI LTS trial relative to those in the pooled GEMINI 1 and 2 data analysis (5.0 [4.4–5.6] vs 7.7 [6.0–9.4]). The incidence rates [95% CI] of pneumonia, bronchopneumonia, primary atypical pneumonia, and lobar pneumonia in vedolizumab-treated patients were 0.9 [0.7–1.2], <0.1 [0.0–0.1], <0.1 [0.0–0.1], and 0.1 [<0.1–0.2] events per 100 patient-years, respectively [Table 2].

In the GEMINI LTS trial, the proportion of patients with reported respiratory infection declined over time: from 24.8% during months 0–12 to 9.6% during months 60–72 for URTI events, and from 5.7% during months 0–12 to 1.1% during months 60–72 for LRTI events [Table 5].

**Table 5.** Incidence of respiratory tract infections by duration of vedolizumab exposure in the GEMINI LTS trial.^a^

MedDRA HLT and preferred term	Duration of vedolizumab exposure, months^b^											
Patients treated	0 to <12 [*n=* 2243]		12 to <24 [*n=* 1583]		24 to <36 [*n=* 1270]		36 to <48 [*n=* 901]		48 to <60 [*n=* 412]		60 to <72 [*n=* 94]	
	*n*	%	*n*	%	*n*	%	*n*	%	*n*	%	*n*	%
URTI AEs	556	24.8	361	22.8	271	21.3	168	18.6	48	11.7	9	9.6
Nasopharyngitis	310	13.8	188	11.9	135	10.6	83	9.2	22	5.3	6	6.4
URTI [preferred term]	157	7.0	102	6.4	84	6.6	54	6.0	16	3.9	2	2.1
Sinusitis	102	4.5	64	4.0	40	3.1	24	2.7	7	1.7	2	2.1
Pharyngitis	29	1.3	26	1.6	16	1.3	9	1.0	3	0.7	0	-
Rhinitis	14	0.6	11	0.7	7	0.6	6	0.7	0	-	0	-
Tonsillitis	10	0.4	11	0.7	4	0.3	2	0.2	0	-	0	-
Laryngitis	7	0.3	5	0.3	4	0.3	4	0.4	2	0.5	0	-
Tracheitis	1	<0.1	1	<0.1	0	-	1	0.1	0	-	0	-
Acute sinusitis	4	0.2	2	0.1	3	0.2	0	-	1	0.2	0	-
Acute tonsillitis	8	0.4	6	0.4	1	<0.1	0	-	1	0.2	0	-
Chronic sinusitis	4	0.2	1	<0.1	1	<0.1	0	-	1	0.2	0	-
Sinobronchitis	0	-	2	0.1	0	-	0	-	0	-	0	-
Tracheobronchitis	1	<0.1	0	-	0	-	0	-	0	-	0	-
Peritonsillar abscess	1	<0.1	0	-	0	-	0	-	0	-	0	-
Pharyngotonsilitis	1	<0.1	0	-	0	-	0	-	0	-	0	-
LRTI AEs	127	5.7	78	4.9	58	4.6	33	3.7	14	3.4	1	1.1
Bronchitis	80	3.6	53	3.3	37	2.9	30	3.3	12	2.9	1	1.1
Pneumonia	22	1.0	15	0.9	10	0.8	2	0.2	1	0.2	0	-
LRTI [preferred term]	27	1.2	13	0.8	9	0.7	1	0.1	1	0.2	0	-
Lobar pneumonia	2	<0.1	0	-	3	0.2	0	-	0	-	0	-
Bronchopneumonia	1	<0.1	0	-	0	-	0	-	0	-	0	-
Lung infection	0	-	1	<0.1	0	-	0	-	0	-	0	-
Primary atypical pneumonia	0	-	0	-	1	<0.1	0	-	0	-	0	-

Except where otherwise noted, LRTI is defined according to the MedDRA HLT ‘lower respiratory tract and lung infection’.

AE, adverse event; HLT, high-level term; LRTI, lower respiratory tract infection; LTS, long-term safety; MedDRA, Medical Dictionary for Regulatory Activities; URTI, upper respiratory tract infection.

^a^Interim data cut-off’: May 19, 2015.

^b^Exposure was calculated as last assessment date first dose date + 1 day. Exposure includes exposure from previous vedolizumab studies.

## 4. Discussion

Our results show that, in the GEMINI 1 and 2 studies, vedolizumab therapy was not associated with significantly higher rates of RTI. Most of the URTIs and LRTIs that occurred in patients receiving vedolizumab were not serious and did not result in treatment discontinuation. Multivariate Cox proportional hazards modelling showed that, unlike smoking or previous exposure to TNF antagonist therapy, vedolizumab therapy was not a risk factor for LRTI events, in either UC or CD. Moreover, analysis of vedolizumab serum concentrations did not reveal any significant association or consistent trends between vedolizumab quartile levels and RTIs, URTIs, or LRTIs [unpublished data]. These results are consistent with a meta-analysis of pooled data from six randomised placebo-controlled clinical trials in patients with IBD, which found no significant differences between vedolizumab and placebo in the risk of all serious infections [relative risk 1.17; 95% CI: 0.51–2.69].^24^

Pneumonia is an event of particular interest, and rates were low in these post hoc analyses, with most classified as non-serious. Of the 11/1434 vedolizumab-treated patients in GEMINI 1 and 2 who had pneumonia, the event was considered serious in two patients. In the GEMINI LTS trial, of the 49/2243 patients who had pneumonia events [0.92 per 100 patient-years], 18 were considered to have serious pneumonia [0.33 per 100 patient-years]. One patient discontinued vedolizumab due to pneumonia. The data suggest that long-term vedolizumab therapy does not have any progressive or cumulative effect on susceptibility to pneumonia. These findings are consistent with the gut-selective mechanism of action of vedolizumab and lack of systemic immunosuppressive effects.^14^ The comparator group comprised patients who responded to placebo and were subsequently maintained on placebo, suggesting a relatively lower disease severity that would, if anything, bias the study toward finding an increase in pneumonia risk with vedolizumab exposure. This finding also provides greater confidence in the conclusion that vedolizumab was not associated with a greater risk of pneumonia. Although previous studies had suggested a potential increased risk of URTI for vedolizumab, based on mucosal vascular addressin cell adhesion molecule 1 [MAdCAM-1] expression in the oropharynx, our results do not support a clinically relevant corollary of this finding.^15^

An increased risk of RTI is one limitation of TNF antagonist therapy.^3,11–13^ Due to their systemic immunosuppressive mode of action, patients exposed to these agents have an increased risk of URTI and LRTI, serious RTIs, and pneumonia-related mortality.^3,11–13^ It is difficult to make an indirect comparison of the absolute rates of URTI and LRTI events observed in this analysis with those from pivotal trials of TNF antagonists in IBD [Supplementary Table 4] for several reasons. First, published reports provide only sparse details regarding these events, and rates of occurrence are expressed as simple proportions rather than exposure-adjusted rates. Second, publications often provide rates for common AEs only—typically those that occurred in >5% of patients. Usually the incidence of LRTIs does not meet this criterion. Consequently, little attention has been drawn to the issue of RTIs in patients receiving TNF antagonists despite observations that LRTIs, and specifically pneumonia, are among the most common serious infections associated with the use of these agents.^3,25^

Risk factors associated with the occurrence of pneumonia include smoking^26^ and TNF antagonist therapy.^3,27–29^ Consistent with this, our analysis identified smoking status, previous use of TNF antagonists, and female sex as independent risk factors for LRTI. In both UC and CD, cigarette smoking was identified as a risk factor for both URTI and LRTI. Although this finding requires little explanation, it is concerning that the prevalence of cigarette smoking remains high in patients with CD. A higher proportion of patients with CD were current smokers compared with those with UC. In GEMINI 1 [UC], 4.2% of females and 7.5% of males were current smokers compared with 29.2% of females and 22.3% of males in GEMINI 2 [CD]. Accordingly, measures to reduce the known risk factors for RTI should be implemented, including referral to smoking cessation programmes, administration of vaccines,^30^ and judicious use of systemic immunosuppression in these patients.^31^

Both the pooled GEMINI 1 and 2 population and patients with UC were more likely to have an LRTI if they had had previous TNF antagonist exposure, despite implementation of a washout period before randomisation^16,17^ [Table 3]. However, there is some evidence to suggest that previous TNF antagonist therapy in patients with rheumatoid arthritis is associated with serious infection,^25^ and switching from a TNF antagonist to a treatment with an alternative mechanism of action may reduce the risks of infection.^32^ An explanation for these observations is not readily apparent. However, patients with previous TNF antagonist exposure may be at greater risk for RTIs because of longer disease duration, slightly greater baseline disease activity, and greater use of additional therapies such as corticosteroids, compared with patients without previous exposure [Supplementary Table 5, available as Supplementary data at *ECCO-JCC* online].

The GEMINI LTS trial provides insight into the risks of long-term exposure to vedolizumab (5430 patient-years to interim analysis date [May 19, 2015]). Rates of URTI and LRTI events were generally lower than those in the GEMINI 1 and 2 studies, and exhibited similar patterns. These lower rates may reflect the improvement in general health in patients responding to long-term vedolizumab therapy. The importance of accruing data from a large number of patients with a long follow-up time, thereby improving sensitivity to rare or delayed safety concerns, should be balanced with the limitations of LTS studies, such as lack of a reference arm. Ongoing registry evaluations, such as the recently initiated ENTYVIO^®^ Outcomes in Real-world Bio-naïve Ulcerative Colitis and Crohn’s Disease Patients [EVOLVE], will provide insight on treatment patterns and outcomes for patients.

In summary, this post hoc analysis of controlled trials and their LTS study provides a comprehensive assessment of LRTIs and URTIs in almost 6500 patient-years of exposure to vedolizumab for the treatment of IBD. Vedolizumab therapy was not associated with an increased incidence of LRTIs compared with placebo.

## Funding

This work was supported by Takeda Pharmaceutical Company Ltd. Medical writing support was provided by Khalid Siddiqui of Chameleon Communications International Ltd, UK [a Healthcare Consultancy Group Company] and sponsored by Takeda Pharmaceutical Company Ltd.

## Conflict of Interest

BGF: consultancy for Abbott/AbbVie, ActoGeniX, Akros, Albireo Pharma, Amgen, AstraZeneca, Avaxia Biologics Inc., Avir Pharma, Axcan, Baxter Healthcare Corp., Biogen, Boehringer Ingelheim, Bristol-Myers Squibb, Calypso Biotech, Celgene, Elan/Biogen, enGene, Ferring Pharmaceuticals, gIcare Pharma, Gilead, Given Imaging Inc., GlaxoSmithKline, Ironwood Pharma, Janssen Biotech [Centocor], JnJ/Janssen, Kyowa Hakko Kirin Co. Ltd, Lexicon, Lilly, Lycera, Merck, Mesoblast Pharma, Millennium, Nektar, Nestlé, Novartis, Novo Nordisk, Pfizer, Prometheus Therapeutics and Diagnostics, Protagonist, Receptos, Roche/Genentech, Salix Pharma, Serono, Shire, Sigmoid Pharma, Synergy Pharma Inc., Takeda, Teva Pharma, TiGenix, Tillotts, UCB, Vertex Pharma, VHsquared, Warner Chilcott, Wyeth, Zealand Pharma, and Zyngenia; grant/research support from Abbott/AbbVie, Amgen, AstraZeneca, Bristol-Myers Squibb, Janssen Biotech [Centocor], JnJ/Janssen, Millennium, Pfizer, Receptos, Roche/Genentech, Sanofi, Santarus, Tillotts, and UCB; member [board of directors] of Robarts Clinical Trials, Inc. FB: employee of Takeda Pharmaceuticals International Co., Cambridge, MA, USA. MK and AB: employees of Takeda International ‑ UK Branch. SPLT: employee of Oxford University Hospitals NHS Foundation Trust and the University of Oxford; grants/research support from AbbVie, IOIBD, Lilly, Norman Collison Foundation, UCB, and Vifor; consulting fees from AbbVie, Amgen, Biogen, Boehringer Ingelheim, Bristol-Myers Squibb, Celgene, ChemoCentryx, Cosmo, Ferring, Giuliani SpA, GlaxoSmithKline, Janssen, Lilly, MSD, Neovacs, Norman Collison Foundation, Novartis, Novo Nordisk, NPS Pharmaceuticals, Pfizer, Proximagen, Receptos, Shire, Sigmoid Pharma, Takeda, Topivert, UCB, VHsquared, and Vifor; speaker fees from AbbVie, Amgen, Biogen, Ferring, and Takeda.

## Author Contributions

BGF, FB, MK, AB, and SPLT have contributed to the study concept and design, acquisition of data, analysis and interpretation of data, drafting of the manuscript, critical revision of the manuscript for important intellectual content, and approving the final submitted draft.

## Supplementary Data

Supplementary data are available at *ECCO-JCC* online.

Supplementary Material

## Notes

Conference presentation: This study was previously presented as a poster at the *European Crohn’s and Colitis Organisation 2017* and at *Digestive Disease Week 2017*

## References

[1] Deepak P, Stobaugh DJ, Ehrenpreis ED Infectious complications of TNF-α inhibitor monotherapy versus combination therapy with immunomodulators in inflammatory bowel disease: analysis of the Food and Drug Administration Adverse Event Reporting System. J Gastrointestin Liver Dis 2013;22:269–76. 24078983

[2] Grijalva CG, Chen L, Delzell E, et al Initiation of tumor necrosis factor-α antagonists and the risk of hospitalization for infection in patients with autoimmune diseases. JAMA 2011;306:2331–9. 22056398 10.1001/jama.2011.1692 PMC3428224

[3] Lichtenstein GR, Feagan BG, Cohen RD, et al Serious infection and mortality in patients with Crohn’s disease: more than 5 years of follow-up in the TREAT™ registry. Am J Gastroenterol 2012;107:1409–22. 22890223 10.1038/ajg.2012.218 PMC3438468

[4] McAuliffe ME, Lanes S, Leach T, et al Occurrence of adverse events among patients with inflammatory bowel disease in the HealthCore Integrated Research Database. Curr Med Res Opin 2015;31:1655–64. 26135040 10.1185/03007995.2015.1065242

[5] Hamzaoglu H, Cooper J, Alsahli M, Falchuk KR, Peppercorn MA, Farrell RJ Safety of infliximab in Crohn’s disease: a large single-center experience. Inflamm Bowel Dis 2010;16:2109–16. 20848473 10.1002/ibd.21290

[6] Hanauer SB, Feagan BG, Lichtenstein GR, et al; ACCENT I Study Group Maintenance infliximab for Crohn’s disease: the ACCENT I randomised trial. Lancet 2002;359:1541–9. 12047962 10.1016/S0140-6736(02)08512-4

[7] Present DH, Rutgeerts P, Targan S, et al Infliximab for the treatment of fistulas in patients with Crohn’s disease. N Engl J Med 1999;340:1398–405. 10228190 10.1056/NEJM199905063401804

[8] Rutgeerts P, Sandborn WJ, Feagan BG, et al Infliximab for induction and maintenance therapy for ulcerative colitis. N Engl J Med 2005;353:2462–76. 16339095 10.1056/NEJMoa050516

[9] Smolen JS, van Vollenhoven R, Kavanaugh A, et al Certolizumab pegol plus methotrexate 5-year results from the rheumatoid arthritis prevention of structural damage [RAPID] 2 randomized controlled trial and long-term extension in rheumatoid arthritis patients. Arthritis Res Ther 2015;17:245. 26353833 10.1186/s13075-015-0767-2 PMC4565002

[10] Sandborn WJ, Feagan BG, Stoinov S, et al; PRECISE 1 Study Investigators Certolizumab pegol for the treatment of Crohn’s disease. N Engl J Med 2007;357:228–38. 17634458 10.1056/NEJMoa067594

[11] Long MD, Martin C, Sandler RS, Kappelman MD Increased risk of pneumonia among patients with inflammatory bowel disease. Am J Gastroenterol 2013;108:240–8. 23295276 10.1038/ajg.2012.406 PMC4624299

[12] Ananthakrishnan AN, McGinley EL Infection-related hospitalizations are associated with increased mortality in patients with inflammatory bowel diseases. J Crohns Colitis 2013;7:107–12. 22440891 10.1016/j.crohns.2012.02.015

[13] D’Haens G, Reinisch W, Colombel JF, et al; ENCORE investigators Five-year safety data from ENCORE, a European observational safety registry for adults with Crohn’s disease treated with infliximab [Remicade®] or conventional therapy. J Crohns Colitis 2017;11:680–9. 28025307 10.1093/ecco-jcc/jjw221

[14] Soler D, Chapman T, Yang LL, Wyant T, Egan R, Fedyk ER The binding specificity and selective antagonism of vedolizumab, an anti-alpha4beta7 integrin therapeutic antibody in development for inflammatory bowel diseases. J Pharmacol Exp Ther 2009;330:864–75. 19509315 10.1124/jpet.109.153973

[15] Wyant T, Fedyk E, Abhyankar B An overview of the mechanism of action of the monoclonal antibody vedolizumab. J Crohns Colitis 2016;10:1437–44. 27252400 10.1093/ecco-jcc/jjw092

[16] Feagan BG, Rutgeerts P, Sands BE, et al; GEMINI 1 Study Group Vedolizumab as induction and maintenance therapy for ulcerative colitis. N Engl J Med 2013;369:699–710. 23964932 10.1056/NEJMoa1215734

[17] Sandborn WJ, Feagan BG, Rutgeerts P, et al; GEMINI 2 Study Group Vedolizumab as induction and maintenance therapy for Crohn’s disease. N Engl J Med 2013;369:711–21. 23964933 10.1056/NEJMoa1215739

[18] Colombel JF, Sands BE, Rutgeerts P, et al The safety of vedolizumab for ulcerative colitis and Crohn’s disease. Gut 2017;66:839–51. 26893500 10.1136/gutjnl-2015-311079 PMC5531223

[19] Sands BE, Feagan BG, Rutgeerts P, et al Effects of vedolizumab induction therapy for patients with Crohn’s disease in whom tumor necrosis factor antagonist treatment failed. Gastroenterology 2014;147:618–27.e3. 24859203 10.1053/j.gastro.2014.05.008

[20] Parikh A, Fox I, Leach T, et al Long-term clinical experience with vedolizumab in patients with inflammatory bowel disease. Inflamm Bowel Dis 2013;19:1691–9. 23591599 10.1097/MIB.0b013e318281f538

[21] Loftus EV Jr, Colombel JF, Feagan BG, et al Long-term efficacy of vedolizumab for ulcerative colitis. J Crohns Colitis 2017;11:400–11. 27683800 10.1093/ecco-jcc/jjw177

[22] Vermeire S, Loftus EV Jr, Colombel JF, et al Long-term efficacy of vedolizumab for Crohn’s disease. J Crohns Colitis 2017;11:412–24. 27683798 10.1093/ecco-jcc/jjw176

[23] Takeda Pharmaceuticals Company Ltd. Data on file. 2018.

[24] Wang MC, Zhang LY, Han W, et al PRISMA–efficacy and safety of vedolizumab for inflammatory bowel diseases: a systematic review and meta-analysis of randomized controlled trials. Medicine [Baltimore] 2014;93:e326. 10.1097/MD.0000000000000326 PMC4603082 25526490

[25] Bykerk VP, Cush J, Winthrop K, et al Update on the safety profile of certolizumab pegol in rheumatoid arthritis: an integrated analysis from clinical trials. Ann Rheum Dis 2015;74:96–103. 24092417 10.1136/annrheumdis-2013-203660 PMC4283674

[26] Almirall J, Bolíbar I, Serra-Prat M, et al; Community-Acquired Pneumonia in Catalan Countries [PACAP] Study Group New evidence of risk factors for community-acquired pneumonia: a population-based study. Eur Respir J 2008;31:1274–84. 18216057 10.1183/09031936.00095807

[27] Curtis JR, Yang S, Patkar NM, et al Risk of hospitalized bacterial infections associated with biologic treatment among US veterans with rheumatoid arthritis. Arthritis Care Res [Hoboken] 2014;66:990–7. 10.1002/acr.22281 PMC5059836 24470378

[28] Kalb RE, Fiorentino DF, Lebwohl MG, et al Risk of serious infection with biologic and systemic treatment of psoriasis: results from the Psoriasis Longitudinal Assessment and Registry [PSOLAR]. JAMA Dermatol 2015;151:961–9. 25970800 10.1001/jamadermatol.2015.0718

[29] Lane MA, McDonald JR, Zeringue AL, et al TNF-α antagonist use and risk of hospitalization for infection in a national cohort of veterans with rheumatoid arthritis. Medicine [Baltimore] 2011;90:139–45. 10.1097/MD.0b013e318211106a PMC3076552 21358439

[30] Farraye FA, Melmed GY, Lichtenstein GR, Kane SV ACG clinical guideline: preventive care in inflammatory bowel disease. Am J Gastroenterol 2017;112:241–58. 28071656 10.1038/ajg.2016.537

[31] Gomollón F, Dignass A, Annese V, et al; ECCO Third European evidence-based consensus on the diagnosis and management of Crohn’s disease 2016. Part 1: diagnosis and medical management. J Crohns Colitis 2017;11:3–25. 27660341 10.1093/ecco-jcc/jjw168

[32] Yun H, Xie F, Delzell E, et al Risk of hospitalised infection in rheumatoid arthritis patients receiving biologics following a previous infection while on treatment with anti-TNF therapy. Ann Rheum Dis 2015;74:1065–71. 24608404 10.1136/annrheumdis-2013-204011 PMC4441344

