# Supplementary material for: Respiratory Tract Infections in Patients With Inflammatory Bowel Disease: Safety Analyses From Vedolizumab Clinical Trials
Source: J Crohns Colitis. 2018 May 17;12(8):905–19. doi: 10.1093/ecco-jcc/jjy047 (PMC6065483; doi:10.1093/ecco-jcc/jjy047)
Supplement: Supplementary Material [file jjy047_suppl_supplementary_material.docx]

Supplementary Material

Supplementary Table 1. MedDRA (version 14.0) search terms

| **High-level term** | **Preferred term** |
| --- | --- |
| Upper respiratory tract infections | Acute sinusitis |
|  | Acute tonsillitis |
|  | Adenoiditis |
|  | Chronic sinusitis |
|  | Chronic tonsillitis |
|  | Epiglottitis |
|  | Epiglottitis obstructive |
|  | Laryngitis |
|  | Laryngotracheitis obstructive |
|  | Nasal abscess |
|  | Nasal vestibulitis |
|  | Nasopharyngitis |
|  | Peritonsillar abscess |
|  | Peritonsillitis |
|  | Pharyngeal abscess |
|  | Pharyngitis |
|  | Pharyngolaryngeal abscess |
|  | Pharyngotonsillitis |
|  | Pseudocroup |
|  | Rhinitis |
|  | Rhinolaryngitis |
|  | Rhinotracheitis |
|  | Sinobronchitis |
|  | Sinusitis |
|  | Thornwaldt disease |
|  | Tonsillitis |
|  | Tracheitis |
|  | Tracheitis obstructive |
|  | Tracheobronchitis |
|  | Tracheostomy infection |
|  | Upper aerodigestive tract infection |
|  | Upper respiratory tract infection |
| Lower respiratory tract and lung infections | Bronchitis |
|  | Bronchopneumonia |
|  | Congenital pneumonia |
|  | Embolic pneumonia |
|  | Infective exacerbation of bronchiectasis |
|  | Infective exacerbation of chronic obstructive airways disease |
|  | Infective pulmonary exacerbation of cystic fibrosis |
|  | Lobar pneumonia |
|  | Lower respiratory tract infection |
|  | Lung abscess |
|  | Lung infection |
|  | Mediastinal abscess |
|  | Miliary pneumonia |
|  | Neonatal pneumonia |
|  | Pleural infection |
|  | Pneumonia |
|  | Pneumonia necrotising |
|  | Pneumonia primary atypical |
|  | Post procedural pneumonia |
|  | Pyopneumothorax |
|  | Pyothorax |
|  | Sputum purulent |
|  | Young's syndrome |

MedDRA, Medical Dictionary for Regulatory Activities

**Supplementary Table 2. Exposure-adjusted incidence rate per 100 patient-years of respiratory tract infections in GEMINI 1, GEMINI 2, and GEMINI OLE**

| **Category** | **GEMINI 1: UC** | | | | **GEMINI 2: CD** | | | | **Pooled GEMINI 1 and GEMINI 2** | | | | **GEMINI OLE: all vedolizumab-treated^a^** | | | | | |
| --- | --- | --- | --- | --- | --- | --- | --- | --- | --- | --- | --- | --- | --- | --- | --- | --- | --- | --- |
| **Safety population** | **Vedolizumab (n=620) TPY=471.9** | | **Placebo (n=149) TPY=81.2** | | **Vedolizumab (n=814) TPY=586.2** | | **Placebo (n=148) TPY=90.1** | | **Vedolizumab (n=1434) TPY=1058.2** | | **Placebo (n=297) TPY=171.3** | | **UC (n=894)**  **TPY=2285.4** | | **CD**  **(n=1349)**  **TPY=3145.0** | | **Total (N=2243)**  **TPY=5430.3** | |
|  | **n (%)** | **IR^b^ (95% CI)** | **n (%)** | **IR^b^ (95% CI)** | **n (%)** | **IR^b^ (95% CI)** | **n (%)** | **IR^b^ (95% CI)** | **n (%)** | **IR^b^ (95% CI)** | **n (%)** | **IR^b^ (95% CI)** | **n (%)** | **IR^b^ (95% CI)** | **n (%)** | **IR^b^ (95% CI)** | **n (%)** | **IR^b^ (95% CI)** |
| **Lower/upper respiratory tract infection** | | | | | | | | | | | | | | | | | | |
| Any AE | 173 (27.9) | 45.9 (38.7–52.9) | 27 (18.1) | 36.9 (22.4–51.3) | 217 (26.7) | 45.9 (39.5–52.2) | 31 (20.9) | 41.7 (25.9–56.8) | 390 (27.2) | 45.9 (41.1–50.5) | 58 (19.5) | 39.3 (28.6–49.7) | 374 (41.8) | 25.6 (22.6–28.4) | 571 (42.3) | 28.7 (26.0–31.3) | 945 (42.1) | 27.4 (25.4–29.3) |
| **Upper respiratory tract infections** | | | | | | | | | | | | | | | | | | |
| Any AE | 155 (25.0) | 40.2  (33.5–46.7) | 23  (15.4) | 30.7  (17.7–43.7) | 184  (22.6) | 37.6  (32.0–43.2) | 27  (18.2) | 35.4  (21.1–49.2) | 339  (23.6) | 38.7  (34.4–43.0) | 50  (16.8) | 33.0  (23.4–42.5) | 344  (38.5) | 22.3  (19.7–24.9) | 513 (38.0) | 24.3  (22.0–26.6) | 857 (38.2) | 23.5  (21.7–25.2) |
| AE resulting in discontinuation | 1 (0.1) | 0.2  (0.0–0.5) | 0 | – | 1^c^  (0.1) | 0.2  (0.0–0.5) | 0 | – | 1^c^  (0.1) | 0.1  (0.0–0.3) | 0 | – | – | – | – | – | – | – |
| Serious AE | 1  (0.1) | 0.2  (0.0–0.5) | 0 | – | 1  (0.1) | 0.2  (0.0–0.5) | 0 | – | 2  (0.1) | 0.2  (0.0–0.5) | 0 | – | 2 (0.2) | 0.1  (0.0–0.2) | 4 (0.3) | 0.1  (0.0–0.3) | 6 (0.3) | 0.1  (<0.1–0.2) |
| Serious AE resulting in discontinuation | 0 | – | 0 | – | 0 | – | 0 | – | 0 | – | 0 | – | 0 | – | 0 | – | 0 | – |
| Death | 0 | – | 0 | – | 0 | – | 0 | – | 0 | – | 0 | – | 0 | – | 0 | – | 0 | – |
| **Lower respiratory tract infections** | | | | | | | | | | | | | | | | | | |
| Any AE | 31  (5.0) | 6.8  (4.4–9.2) | 7  (4.7) | 9.0  (2.3–15.6) | 48  (5.9) | 8.5  (6.1–10.9) | 7  (4.7) | 8.0  (2.0–14.0) | 79  (5.5) | 7.7  (6.0–9.5) | 14  (4.7) | 8.5  (4.0–12.9) | 87 (9.7) | 4.1  (3.3–5.0) | 161 (11.9) | 5.7  (4.8–6.6) | 248 (11.1) | 5.0  (4.4–5.7) |
| AE resulting in discontinuation | 0 | – | 0 | – | 2^d^  (0.2) | 0.3  (0.0–0.8) | 0 | – | 2^d^  (0.1) | 0.2  (0.0–0.5) | 0 | – | 0 | – | 0 | – | 0 | – |
| Serious AE | 2  (0.3) | 0.4  (0.0–1.0) | 0 | – | 3  (0.4) | 0.5  (0.0–1.1) | 1  (0.7) | 1.1  (0.0–3.3) | 5  (0.3) | 0.5  (0.1–0.9) | 1  (0.3) | 0.6  (0.0–1.7) | 10  (1.1) | 0.4  (0.2–0.7) | 10 (0.7) | 0.3  (0.1–0.5) | 20 (0.9) | 0.4  (0.2–0.5) |
| Serious AE resulting in discontinuation | 0 | – | 0 | – | 1  (0.1) | 0.2  (0.0–0.5) | 0 | – | 1  (0.1) | 0.1  (0.0–0.3) | 0 | – | 0 | – | 0 | – | 0 | – |
| Death | 0 | – | 0 | – | 0 | – | 1  (0.7) | 1.1  (0.0–3.3) | 0 | – | 1^e^  (0.3) | 0.6  (0.0–1.7) | 0 | – | 0 | – | 0 | – |

Lower respiratory tract infections are defined according to the MedDRA high level term ‘lower respiratory tract and lung infection’

^a^Interim data cut-off: May 19, 2015

**^b^**IR, exposure-adjusted incidence rate per 100 patient-years ([number of patients experiencing an AE of interest/total patient exposure time in years] × 100)

^c^Patient with laryngitis

^d^One patient with bronchitis and one with pneumonia

^e^Patient receiving placebo died due to bronchopneumonia, a serious AE

AE, adverse event; CD, Crohn’s disease; IR, incident rate; MedDRA, Medical Dictionary for Regulatory Activities, OLE, open-label extension; TPY, total number of patient-years of exposure; UC, ulcerative colitis

Supplementary Table 3. Summary of lower and upper respiratory tract infections treatment emergent adverse events, time-adjusted rates in GEMINI 1, GEMINI 2, and pooled GEMINI 1 and GEMINI 2 (Safety Population)

| **Category** | **GEMINI 1: UC** | | **GEMINI 2: CD** | | **Pooled GEMINI 1 and GEMINI 2** | |
| --- | --- | --- | --- | --- | --- | --- |
| **Safety population** | **Vedolizumab Q4W (n=498) TPY=368.9** | **Vedolizumab Q8W (n=122) TPY=103.1** | **Vedolizumab Q4W (n=660) TPY=468.7** | **Vedolizumab Q8W (n=154) TPY=117.6** | **Vedolizumab Q4W (n=1158) TPY=837.5** | **Vedolizumab Q8W (n=276) TPY=220.7** |
| **Upper respiratory tract infections, n (Rate per 100 PY)** | | | | | | |
| Any AE | 120 (39.8) | 35 (41.2) | 149 (38.2) | 35 (35.2) | 269 (38.9) | 70 (38.0) |
| Drug-related AE | 24 (6.7) | 8 (8.1) | 43 (9.6) | 7 (6.2) | 67 (8.4) | 15 (7.1) |
| AE resulting in discontinuation | 0 | 0 | 1 (0.2) | 0 | 1 (0.1) | 0 |
| Serious AE | 1 (0.3) | 0 | 1 (0.2) | 0 | 2 (0.2) | 0 |
| Serious infection AE | 1 (0.3) | 0 | 1 (0.2) | 0 | 2 (0.2) | 0 |
| Drug-related serious AE | 0 | 0 | 1 (0.2) | 0 | 1 (0.1) | 0 |
| **Lower respiratory tract infections, n (Rate per 100 PY)** | | | | | | |
| Any AE | 23 (6.4) | 8 (8.0) | 42 (9.4) | 6 (5.2) | 65 (8.1) | 14 (6.5) |
| Drug-related AE | 4 (1.1) | 2 (2.0) | 14 (3.0) | 2 (1.7) | 18 (2.2) | 4 (1.8) |
| AE resulting in discontinuation | 0 | 0 | 2 (0.4) | 0 | 2 (0.2) | 0 |
| Serious AE | 0 | 2 (2.0) | 3 (0.6) | **0** | 3 (0.4) | 2 (0.9) |
| Serious infection AE | 0 | 2 (2.0) | 3 (0.6) | 0 | 3 (0.4) | 0 |
| Drug-related serious AE | 0 | 1 (1.0) | 2 (0.4) | 0 | 2 (0.2) | 1 (0.5) |
| Serious AE resulting in discontinuation | 0 | 0 | 1 (0.1) | 0 | 1 (0.1) | 0 |

Supplementary Table 4. Lower and upper respiratory tract infections in phase 3 trials (and corresponding open-label extensions) of TNF antagonists in adults with Crohn’s disease or ulcerative colitis

| **Reference** | **Trial name**  **Duration**  **Phase** | **Rates of any types of lower respiratory tract infections and upper respiratory tract infections** | |
| --- | --- | --- | --- |
|  |  | **Placebo or other comparator** | **TNF antagonists** |
| **Infliximab in Crohn’s disease** | | | |
| Hanauer *et al*. 2002 | ACCENT I  54 weeks  Phase 3 | n=188   - No rates provided for any respiratory infections | n=193; 5 mg/kg at 2 and 6 weeks and then every 8 weeks   - No rates provided for any respiratory infections   n=192; 5 mg/kg at 2 and 6 weeks and then 10 mg/kg   - No rates provided for any respiratory infections |
| Colombel *et al.* 2010 | SONIC  54 weeks  Phase 3 | Azathioprine n=161; 2.5 mg dose   - Nasopharyngitis:12.4%   Serious infections not specified by type | Infliximab n=163; 5 mg/kg dose   - Nasopharyngitis:9.2%   Serious infections not specified by type |
| Sands *et al*. 2004 | ACCENT II: fistulizing CD  54 weeks  Phase 3 | n=144  Infections not specified by type | n=138; 5 mg/kg  Infections not specified by type |
| **Infliximab in ulcerative colitis** | | | |
| Rutgeerts *et al.* 2005 | ACT 1  54 weeks  Phase 3 | n=121   - URTI (serious): 23.1% (0.8%) - Pharyngitis (serious): 8.3% (0.8%) - Sinusitis (serious): 3.3% (0.8%) - Pneumonia (serious): 0% (0%) - Pleurisy serious: 0% | n=121; 5 mg/kg dose   - URTI (serious): 16.5% (0%) - Pharyngitis (serious): 9.9% (0%) - Sinusitis (serious): 6.6% (0%) - Pneumonia (serious): 1.7% (0%) - Pleurisy serious: 0% |
| Rutgeerts *et al.* 2005 | ACT 2  30 weeks  Phase 3 | n=123   - URTI (serious): 11.4% (0%) - Pharyngitis (serious): 2.4% (0%) - Sinusitis (serious): 5.7% (0%) - Pneumonia (serious): 0% (0%) - Pleurisy serious: 0% | n=121; 5 mg/kg dose   - URTI (serious): 13.2% (0%) - Pharyngitis (serious): 5.8% (0%) - Sinusitis (serious): 9.1% (0%) - Pneumonia (serious): 0% (0%) - Pleurisy serious: 0% |
| **Adalimumab in Crohn’s disease** | | | |
| Hanauer *et al.* 2006 | CLASSIC I  4 weeks  Phase 3 | n=74   - Nasopharyngitis: 1% - Pharyngitis: 3% - Pneumonia: 1% | n=74; 40 mg/20 mg dose induction regimen   - Nasopharyngitis: 3% - Pharyngitis: 1%   n=75; 80 mg/40 mg induction regimen   - Nasopharyngitis: 5% - Pharyngitis: 1%   n=76;160 mg/80 mg induction regimen   - Nasopharyngitis: 5% - Pharyngitis: 7%   All dose regimens   - Pneumonia (serious): 3% (1%) in 160 mg/80 mg group |
| Sandborn *et al*. 2007a | CLASSIC II  56 Weeks  Open-label extension | No placebo for open-label extension component of trial | n=221; 40 mg every other week   - Nasopharyngitis: 17% - Sinusitis: 9% - No rates provided for any LRTI   Serious infections (4%), not specified by type |
| Sandborn *et al.* 2007b | GAIN – CD II  4 weeks  Phase 3 | n=166   - Infection AEs (24% all and 2% serious) not specified by type | n=159; 160/80 mg induction regimen   - Infection AEs (16% all and 0% serious) not specified by type - Rates of non-serious infections including nasopharyngitis and URTIs were similar to placebo |
| Colombel *et al.* 2007 | CHARM – CD III: 56 weeks  Phase 3 | n=261; maintenance   - Nasopharyngitis: 6.9% - Serious pneumonia: 0% | n=260; maintenance 40 mg every other week   - Nasopharyngitis: 11.2% - Serious pneumonia: 0% - No rates provided for any other LRTIs   n=257; maintenance 40 mg weekly   - Nasopharyngitis: 12.1% - Serious pneumonia: 0.4% - No rates provided for any other LRTIs |
| **Adalimumab in ulcerative colitis** | | | |
| Reinisch *et al.* 2011 | ULTRA 1  8 weeks  Phase 3 | n=223  Only serious infections specified by type   - Serious pneumonia: 0.4% | n=130; 80/40 mg and n=223;160/80 mg  Only serious infections specified by type   - No serious RTIs |
| Sandborn *et al*. 2012 | ULTRA 2  52 weeks  Phase 3 | n=260  No rates provided for any respiratory infections | n=257; 40 mg every other week post induction  No rates provided for any respiratory infections |
| Colombel *et al*. 2014 | ULTRA 3  Open-label extension of phase 3 up to 208 weeks | No placebo: open-label extension trial | n=588; 40 mg every other week or weekly (depending upon qualifying trial and response)  No rates provided for any respiratory infections |
| **Golimumab in ulcerative colitis** | | | |
| Sandborn *et al.* 2014a | PURSUIT SC  6 weeks  Integrated phase 2/phase 3 | n=330   - Nasopharyngitis: 3.3% - No rates provided for any LRTIs - Serious pneumonia: 0.1% | n=734; all doses (100/50 mg, 200/100 mg and 400/200 mg)   - Nasopharyngitis: 2.9% - Serious pneumonia: 0.3% |
| Rutgeerts *et al.* 2015 | PURSUIT IV  6 weeks  Integrated phase 2/phase 3 | n=77   - Pharyngitis 0%   Serious infections 0% | n=213; all doses (1, 2, and 4 mg/kg induction regimens)   - Pharyngitis 1.9%   Serious infections 1.9%: none were RTIs |
| Sandborn *et al.* 2014a | PURSUIT Maintenance 54 weeks  Phase 3 | n=156   - URTI: 2.6% - Nasopharyngitis: 7.1% - Pharyngitis: 2.6%   Infections and serious infections not specified by type | n=384; all golimumab (including dose adjusted)   - URTI: 6.8% - Nasopharyngitis: 12.0% - Pharyngitis: 4.2%   Infections and serious infections not specified by type |
| **Certolizumab in Crohn’s disease** | | | |
| Sandborn *et al.* 2007b | PRECISE 1  26 weeks  Phase 3 | n=329   - Nasopharyngitis: 8%   Serious infection AEs (<1%): none were RTIs | n=331; 400 mg dose   - Nasopharyngitis: 13% (*P*=0.04)   Serious infection AEs (2%): none were RTIs |
| Schreiber *et al.* 2007 | PRECISE 2  26 weeks  Phase 3 | n=212; maintenance phase   - Nasopharyngitis: 4% - Serious pneumonia: 0% - Serious lobar pneumonia: 0% | n=216; maintenance phase, 400 mg dose   - Nasopharyngitis: 6% - Serious pneumonia: <1% - Serious lobar pneumonia: 0% |
| Sandborn *et al.* 2014b | PRECISE 3  Up to 18 months  Open-label extension to phase 3 | No placebo: open-label extension trial | n=595; 400 mg every 4 weeks  Nasopharyngitis: 15.3% |
| Sandborn *et al.* 2010 | PRECISE 4  52 weeks  Open-label extension to phase 3 | No placebo: open-label extension trial | n=124; 400 mg dose  Infections and serious infections not specified by type |

*The rate of any respiratory tract infection is summarized as provided in the publication. Absence of data for respiratory tract infection does not necessarily indicate a zero rate because publications may report only the most common AEs

AE, adverse event; CD, Crohn’s disease; LRTI, lower respiratory tract infection; RTI, respiratory tract infection; TNF, tumor necrosis factor; URTI, upper respiratory tract infection

**Supplementary Table 5. Demographics and baseline characteristics for patients receiving vedolizumab in the GEMINI 1 and GEMINI 2 trials stratified by prior TNF antagonist exposure**

|  | **GEMINI 1: UC**  **N = 620** | | **GEMINI 2: CD**  **N = 814** | | **Pooled GEMINI 1 and 2**  **N = 1434** | |
| --- | --- | --- | --- | --- | --- | --- |
| **Characteristic** | **Prior TNF**  **n = 311** | **TNF naïve**  **n = 309** | **Prior TNF**  **n = 535** | **TNF naïve**  **n = 279** | **Prior TNF**  **N = 846** | **TNF naïve**  **N = 588** |
| Age, mean (SD) years^a^ | 39.7 (12.9) | 40.5 (13.3) | 35.9 (11.8) | 34.7 (12.1) | 37.3 (12.3) | 37.7 (13.0) |
| Female sex, n (%) | 122 (39.2) | 134 (43.4) | 303 (56.6) | 132 (47.3) | 425 (50.2) | 266 (45.2) |
| White, n (%) | 287 (92.3) | 231 (74.8) | 500 (93.5) | 231 (82.8) | 787 (93.0) | 462 (78.6) |
| Body weight, mean (SD) kg | 75.2 (17.8) | 71.6 (18.6) | 71.8 (19.8) | 66.9 (19.4) | 73.1 (19.1) | 69.3 (19.1) |
| Baseline disease activity, mean (SD) CDAI score^b^ | – | – | 327.2 (68.52) | 315.1 (67.76) | 327.2 (68.52) | 315.1 (67.76) |
| Baseline common disease activity score,^c^ mean (SD) | 6.1 (1.61) | 6.0 (1.59) | 5.8 (1.75) | 5.2 (1.70) | 5.9 (1.70) | 5.6 (1.68) |
| On-study surgery, n (%) | 39 (12.5) | 10 (3.2) | 90 (16.8) | 36 (12.9) | 129 (15.2) | 46 (7.8) |
| Duration of disease, mean (SD) years^d^ | 7.16 (6.10) | 6.15 (5.96)^e^ | 10.50 (7.43) | 6.33 (6.98) | 9.27 (7.15) | 6.24 (6.46)^f^ |
| Disease duration <7 years, n (%) | 189 (60.8) | 217 (70.2) | 204 (38.1) | 191 (68.5) | 393 (46.5) | 408 (69.4) |
| Disease duration ≥7 years, n (%) | 122 (39.2) | 92 (29.8) | 331 (61.9) | 88 (31.5) | 453 (53.5) | 180 (30.6) |
| Any prior TNF antagonist failure, n (%) | 261 (83.9) | 5 (1.6) | 491 (91.8) | 6 (2.2) | 752 (88.9) | 11 (1.9) |
| Prior immunomodulator, n (%) | 263 (84.6) | 209 (67.6) | 478 (89.3) | 209 (74.9) | 741 (87.6) | 418 (71.1) |
| Prior corticosteroid, n (%) | 306 (98.4) | 299 (96.8) | 514 (96.1) | 263 (94.3) | 820 (96.9) | 562 (95.6) |
| Concomitant narcotic use, n (%) | 81 (26.0) | 42 (13.6) | 228 (42.6) | 48 (17.2) | 309 (36.5) | 90 (15.3) |
| Concomitant immunomodulator, n (%) | 41 (13.2) | 73 (23.6) | 80 (15.0) | 53 (19.0) | 121 (14.3) | 126 (21.4) |
| Concomitant corticosteroid, n (%) | 114 (36.7) | 112 (36.2) | 194 (36.3) | 86 (30.8) | 308 (36.4) | 198 (33.7) |
| Concomitant corticosteroid and immunomodulator, n (%) | 50 (16.1) | 49 (15.9) | 74 (13.8) | 63 (22.6) | 124 (14.7) | 112 (19.0) |
| Former smoker, n (%) | 114 (36.7) | 90 (29.1) | 131 (24.5) | 59 (21.1) | 245 (29.0) | 149 (25.3) |
| Current smoker, n (%) | 17 (5.5) | 19 (6.1) | 143 (26.7) | 73 (26.2) | 160 (18.9) | 92 (15.6) |
| Elevated baseline c-reactive protein, n (%) | – | – | 447 (83.6) | 224 (80.3) | 447 (52.8) | 224 (38.1) |
| Baseline fecal calprotectin, mean (SD) | 1827.8 (2742.18) | 1601.2 (2385.42) | 1289.9 (2059.23) | 1160.6 (1748.43) | 1487.4 (2346.09) | 1392.9 (2118.07) |

Prior lung disease was not a specific exclusion criterion.

For GEMINI 1 and GEMINI 2 the baseline was Week 0 of each study.

^a^For the GEMINI LTS trial, age was defined as (1 + first dose date in the GEMINI LTS trial-birth date)/365.25.

^b^CDAI was not collected for de novo patients.

^c^Baseline disease activity scores based on partial Mayo Score for GEMINI 1 (UC), HBI score for GEMINI 2 (CD), and common index for pooled GEMINI 1 and 2. Common index ranged from 0 to 9 to allow the combination of baseline partial Mayo and HBI scores in the pooled analysis.

^d^For the GEMINI LTS study data, disease duration defined as (1 + first dose date in the GEMINI LTS trial - diagnosis date)/365.25.

^e^*n =* 307.

^f^*n =* 586.

CD, Crohn’s disease; CDAI, Crohn’s Disease Activity Index; HBI, Harvey–Bradshaw Index; LTS, long-term safety; SD, standard deviation; TNF, tumour necrosis factor; UC, ulcerative colitis.

**References**

Colombel JF, Sandborn WJ, Rutgeerts P *et al.* Adalimumab for maintenance of clinical response and remission in patients with Crohn's disease: the CHARM trial. Gastroenterology 2007;132:52-65.

Colombel JF, Sandborn WJ, Reinisch W *et al*. Infliximab, azathioprine, or combination therapy for Crohn's disease. N Engl J Med 2010;362:1383-95.

Colombel JF, Sandborn WJ, Ghosh S *et al*. Four-year maintenance treatment with adalimumab in patients with moderately to severely active ulcerative colitis: Data from ULTRA 1, 2, and 3. Am J Gastroenterol 2014;109:1771-80.

Hanauer SB, Feagan BG, Lichtenstein GR *et al*. Maintenance infliximab for Crohn's disease: the ACCENT I randomised trial. Lancet 2002;359:1541-9.

Hanauer SB, Sandborn WJ, Rutgeerts P *et al.* Human anti-tumor necrosis factor monoclonal antibody (adalimumab) in Crohn's disease: the CLASSIC-I trial. Gastroenterology 2006;130:323-33.

Reinisch W, Sandborn WJ, Hommes DW *et al.* Adalimumab for induction of clinical remission in moderately to severely active ulcerative colitis: results of a randomised controlled trial. Gut 2011;60:780-7.

Rutgeerts P, Sandborn WJ, Feagan BG *et al.* Infliximab for induction and maintenance therapy for ulcerative colitis. N Engl J Med 2005;353:2462-76.

Rutgeerts P, Feagan BG, Marano CW *et al.* Randomised clinical trial: a placebo-controlled study of intravenous golimumab induction therapy for ulcerative colitis. Aliment Pharmacol Ther 2015;42:504-14.

Sandborn WJ, Feagan BG, Stoinov S *et al.* Certolizumab pegol for the treatment of Crohn's disease. N Engl J Med 2007;357:228-38.

Sandborn WJ, Hanauer SB, Rutgeerts P *et al.* Adalimumab for maintenance treatment of Crohn's disease: results of the CLASSIC II trial. Gut 2007;56:1232-9.

Sandborn WJ, Rutgeerts P, Enns R *et al.* Adalimumab induction therapy for Crohn disease previously treated with infliximab: a randomized trial. Ann Intern Med 2007;146:829-38.

Sandborn WJ, Schreiber S, Hanauer SB *et al.* Reinduction with certolizumab pegol in patients with relapsed Crohn's disease: results from the PRECiSE 4 Study. Clin Gastroenterol Hepatol 2010;8:696-702.

Sandborn WJ, van Assche G, Reinisch W *et al.* Adalimumab induces and maintains clinical remission in patients with moderate-to-severe ulcerative colitis. Gastroenterology 2012;142:257-65.

Sandborn WJ, Lee SD, Randall C *et al*. Long-term safety and efficacy of certolizumab pegol in the treatment of Crohn's disease: 7-year results from the PRECiSE 3 study. Aliment Pharmacol Ther 2014;40:903-16.

Sandborn WJ, Feagan BG, Marano C *et al*. Subcutaneous golimumab induces clinical response and remission in patients with moderate-to-severe ulcerative colitis. Gastroenterology 2014;146:85-95.

Sandborn WJ, Feagan BG, Marano C *et al*. Subcutaneous golimumab maintains clinical response in patients with moderate-to-severe ulcerative colitis. Gastroenterology 2014;146:96-109.

Sands BE, Blank MA, Patel K *et al*. Long-term treatment of rectovaginal fistulas in Crohn's disease: response to infliximab in the ACCENT II Study. Clin Gastroenterol Hepatol 2004;2:912-20.

Schreiber S, Khaliq-Kareemi M, Lawrance IC *et al.* Maintenance therapy with certolizumab pegol for Crohn's disease. N Engl J Med 2007;357:239-50.
